# Hypoxia increases genome-wide bivalent epigenetic marking by specific gain of H3K27me3

**DOI:** 10.1186/s13072-016-0086-0

**Published:** 2016-10-26

**Authors:** Peggy Prickaerts, Michiel E. Adriaens, Twan van den Beucken, Elizabeth Koch, Ludwig Dubois, Vivian E. H. Dahlmans, Caroline Gits, Chris T. A. Evelo, Michelle Chan-Seng-Yue, Bradly G. Wouters, Jan Willem Voncken

**Affiliations:** 1 Department of Molecular Genetics, Maastricht University Medical Centre, Maastricht, The Netherlands; 2 Department of Bioinformatics (BiGCaT), Maastricht University Medical Centre, Maastricht, The Netherlands; 3 Maastricht Centre for Systems Biology (MaCSBio), Maastricht University Medical Centre, Maastricht, The Netherlands; 4 Maastricht Radiation Oncology (MaastRO) Laboratory, Maastricht University Medical Centre, Maastricht, The Netherlands; 5 Princess Margaret Cancer Centre and Campbell Family Institute for Cancer Research, University Health Network, Toronto, ON Canada; 6 Department of Medical Biophysics, University of Toronto, Toronto, ON Canada; 7 Informatics and Bio-computing Program, Ontario Institute for Cancer Research, Toronto, ON Canada; 8 Department of Radiation Oncology, University of Toronto, Toronto, ON Canada

**Keywords:** Hypoxia, Reoxygenation, Tumor plasticity, Cancer stemness, Bivalent marking, H3K27me3, H3K4me3, KDM, Histone demethylase, Chromatin immunoprecipitation, Deep sequencing

## Abstract

**Background:**

Trimethylation at histone H3 lysine 4 (H3K4me3) and lysine 27 (H3K27me3) controls gene activity during development and differentiation. Whether H3K4me3 and H3K27me3 changes dynamically in response to altered microenvironmental conditions, including low-oxygen conditions commonly present in solid tumors, is relatively unknown. Demethylation of H3K4me3 and H3K27me3 is mediated by oxygen and 2-oxoglutarate dioxygenases enzymes, suggesting that oxygen deprivation (hypoxia) may influence histone trimethylation. Using the MCF7 breast epithelial adenocarcinoma cell model, we have determined the relationship between epigenomic and transcriptomic reprogramming as a function of fluctuating oxygen tension.

**Results:**

We find that in MCF7, H3K4me3 and H3K27me3 marks rapidly increase at specific locations throughout the genome and are largely reversed upon reoxygenation. Whereas dynamic changes are relatively highest for H3K27me3 marking under hypoxic conditions, H3K4me3 occupation is identified as the defining epigenetic marker of transcriptional control. In agreement with the global increase of H3K27 trimethylation, we provide direct evidence that the histone H3K27me3 demethylase KDM6B/JMJD3 is inactivated by limited oxygen. In situ immunohistochemical analysis confirms a marked rise of histone trimethylation in hypoxic tumor areas. Acquisition of H3K27me3 at H3K4me3-marked loci results in a striking increase in “bivalent” epigenetic marking. Hypoxia-induced bivalency substantially overlaps with embryonal stem cell-associated genic bivalency and is retained at numerous loci upon reoxygenation. Transcriptional activity is selectively and progressively dampened at bivalently marked loci upon repeated exposure to hypoxia, indicating that this subset of genes uniquely maintains the potential for epigenetic regulation by KDM activity.

**Conclusions:**

These data suggest that dynamic regulation of the epigenetic state within the tumor environment may have important consequences for tumor plasticity and biology.

**Electronic supplementary material:**

The online version of this article (doi:10.1186/s13072-016-0086-0) contains supplementary material, which is available to authorized users.

## Background

Cancer cells in solid tumors are often exposed to repeated cycles of oxygen deprivation and reoxygenation that result from inadequate blood supply due to poorly developed vasculature [1]. Tumor oxygenation in situ is known to reach very low values, and repeated oxygen deprivation and reoxygenation promote tumor stem cell properties, metastasis, and negatively affect patient prognosis as adaptive responses to hypoxia severely decrease efficacy of both ionizing radiation and chemotherapy [2–7]. Hypoxia has also been shown to stimulate reprogramming and establishment of induced pluripotent stem cells [8–11]. Transcriptional modulation of genes involved in glycolysis, angiogenesis, pH homeostasis and apoptosis (i.e., anti-apoptotic genes) enables cancer cells to adapt to and survive in the hypoxic tumor environment. Transcriptional changes in hypoxic cancer cells are controlled by several well-understood hypoxia response pathways [12–14]. Stabilization of HIF1A under low-oxygen conditions is obtained through interruption of prolyl 4-hydroxylase (PHD)-mediated hydroxylation of HIF1A and subsequent HIF1A ubiquitylation by the von Hippel–Lindau tumor suppressor (pVHL) E3 ligase complex [15–17]. PHDs belong to a larger family of 2-oxoglutarate-dependent dioxygenases (2-OGDO), including histone demethylases, whose activity is controlled by oxygen, iron and 2-oxoglutarate (2-OG; α-ketoglutarate) [18–20].

The ability of cells to respond to their microenvironment is mediated by epigenetic regulatory mechanisms; as such, phenotypic plasticity constitutes a major underlying mechanism in development, maintenance of homeostasis and cellular diversity [21, 22]. Epigenetic control is coordinated at the level of DNA, by (hydroxy)methylation, RNA, through involvement of regulatory noncoding RNAs, and protein, i.a., by (in)activation of epigenetic regulators and covalent posttranslational modification of histones [23]. Their concerted action affects access of transcriptionally regulatory proteins to chromatin by nucleosome repositioning, recruitment of regulatory factors (i.e., scaffolding factors) and/or altered chromatin compaction. These principles also apply to other DNA-templated processes, including replication and repair [24, 25]. Histone phosphorylation, acetylation and methylation are examples of covalent chemical modifications that occur at the N-terminal tails of core histones that constitute the nucleosomal units in chromatin [25]. A number of histone trimethylation states, among which H3K4me3, are linked to active gene expression, whereas the relationship between H3K27me3 and transcriptional status appears less unambiguous [24, 25]. H3K27me3 and H3K4me3 states have been intensively studied in the context of transcriptional regulation during lineage commitment, cell fate acquisition and maintenance during development [26–29]. H3K27 is trimethylated by the histone lysine methyl transferases (KMTs) EZH2 and EZH1, as part of the Polycomb repressive complex 2 (PRC2) [30, 31]. H3K4me3 marks are generated by KMT2A-D (MLL1-4) KMTs that belong to the Trithorax Group (TrxG) of epigenetic modifiers [32, 33]. Conversely, H3K27 demethylation is carried out by the histone lysine demethylases (KDMs) KDM6A (UTX) and KDM6B (JMJD3) [34], whereas the KDMs KDM5A-D (JARID1A-D) catalyze H3K4 demethylation [35]. Although in terminally differentiated cells most H3K4me3 and H3K27me3 marks appear mutually exclusive, in embryonic stem cells (ESC) bivalent epigenetic marking is believed to prime key developmental control genes for context-dependent (in)activation [26–29, 36, 37]. Current knowledge on the distribution dynamics of H3K27me3 and H3K4me3 beyond embryonic development is relatively limited.

The Jumonji C-terminal domain histone demethylases, including the H3K27me3 demethylases KDM6A and B, are part of the 2-OGDO family proteins which utilize oxygen as a co-substrate during enzymatic demethylation [18, 20]. This suggests the possibility that histone trimethylation states may be directly coupled to cell oxygenation. Hypoxia has been reported to induce global changes in histone methylation and is known to induce major transcriptomic changes [38, 39]. We recently reported that hypoxia causes repression of DICER through acquisition of H3K27me3 at the DICER locus [40]. However, a systematic profiling of H3K27me3 and H3K4me3 in relation to oxygen deprivation and the consequences thereof for gene transcription is lacking. We therefore studied the dynamics of histone methylation in the context of oxygen deprivation using genome-wide chromatin immunoprecipitation followed by deep sequencing (ChIP-seq) and combined this with expression microarray analysis in MCF7 cells.

We report that the histone H3K27me3 demethylase KDM6B/JMJD3 is inactivated by limited oxygen and show a consequential rapid increase of genomic H3K4me3 and H3K27me3 markings in vitro and in vivo. Acquisition of H3K27me3 at H3K4me3 marking increases “bivalent” epigenomic marking, which displays overlap with embryonal stem cell-associated genic bivalency. Hypoxia-induced bivalent marking is retained at numerous loci upon reoxygenation and selectively and progressively dampens transcriptional activity at these loci upon repeated hypoxic exposure and reoxygenation, indicating that this subset of genes uniquely maintains the potential for epigenetic regulation by KDM activity.

## Results

### Increased hypoxia-induced histone H3 methylation in vivo and in vitro

**Fig. 1:**
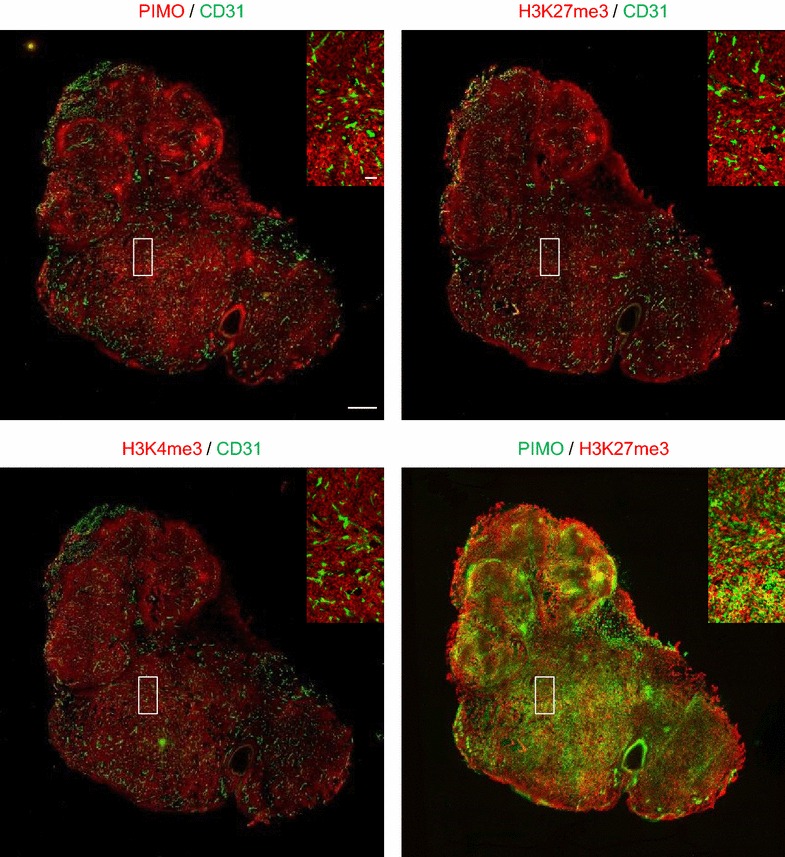
Tumor hypoxia increases trimethylation at H3K4 and H3K27. Representative images of whole tumor parallel sections stained for pimonidazole (hypoxia marker, *red*) and CD31 (PECAM-1; endothelial cell surface marker, *green*; *upper left*), H3K27me3 (*red*) and CD31 (*green*; *upper right*), H3K4me3 (*red*) and CD31 (*green*; *lower left*); *scale bar* represents 1 mm. The *color*-*merged image* (*lower right*) shows pimonidazole (*green*) and H3K27me3 (*red*). *Captions* show enlargements of the indicated areas; *scale bar* represents 100 µm

Based on their structural relatedness to 2-OG-dependent dioxygenases, we hypothesized that the catalytic activity of histone H3K27me3 and H3K4me3 demethylases would be inhibited under hypoxic conditions [18, 20]. To determine whether hypoxia in the tumor environment increases histone H3 trimethylation, we established xenografts of a human breast cancer cell line and analyzed the spatial relationship between hypoxia, vascularization and H3K27me3 and H3K4me3 marks using fluorescence immunohistochemistry. Tumor hypoxia (i.e., pimonidazole uptake) showed an inverse correlation with blood vessel density, as determined by CD31/PECAM-1 co-staining (Fig. 1). Parallel tumor sections stained for pimonidazole and H3K27me3 showed enhanced histone H3K27 trimethylation in hypoxic areas; a merged multi-fluorescence image clearly revealed overlap between hypoxia- and H3K27me3-stained areas within the tumor (Fig. 1). H3K4me3-positive cells appeared to be more evenly distributed over the tumor compared to H3K27me3 and pimonidazole, yet showed good correspondence with pimonidazole-positive areas. Thus, both H3K4 and H3K27 trimethylation are enhanced in hypoxic tumors.

**Fig. 2:**
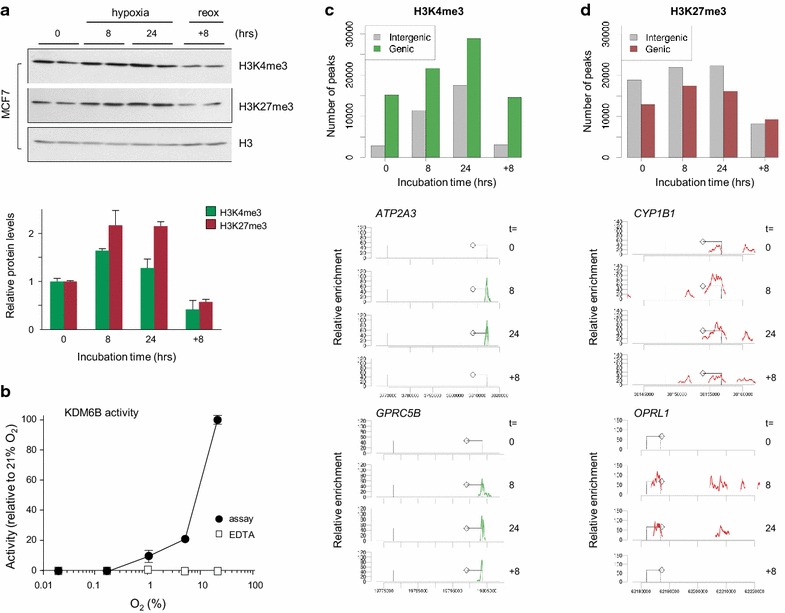
Reversible, oxygen-dependent global changes of H3K4me3 and H3K27me3 levels. **a** Immunoblot detection (IB) of epigenetic changes (H3K4me3 and H3K27me3) in MCF7 cells; loading control: total H3 (H3); *lower panel* quantification. **b** Ability of recombinant JMJD3 to demethylate H3K27me3 peptide in vitro was determined at 0.02, 0.2, 1, 5 and 21% (ambient) partial O_2_ pressure; EDTA was used as a negative control as it blocks JMJD3 activity. Number of peaks for H3K4me3 (**c**) and H3K27me3 (**d**) significantly above background level (*p* < 0.05) at different time points under hypoxic conditions (*t* = 8, *t* = 24 h) and after reoxygenation (*t* = +8 h). *Bottom panels* to **c** and **d**: representative gene tracks of loci displaying H3K4me3 gain (*ATP2A3*, *GPRC5B*) or H3K27me3 gain (*CYP1B1*, *OPRL1*); *diamond symbol* indicates direction of gene transcription

Human tumors are known to contain cells ranging from normal oxygenation to complete anoxia. There is strong evidence that transient changes in blood perfusion contribute to hypoxia in tumors: Vessel occlusion leads to fluctuating oxygen availability in situ (i.e., hypoxia and reoxygenation) and can cause complete anoxia of large numbers of cells [41]. As low oxygen percentage (<0.05% O_2_) induces significantly more treatment resistance than higher oxygen percentages (>1% O_2_) in vitro [42], we set out to examine global histone trimethyl states of H3K4 and H3K27 in MCF7 cells under low oxygen (<0.02% O_2_); to monitor epigenomic effects of fluctuating oxygen, H3K4me3 and H3K27me3 levels were also monitored in response to reoxygenation (8 h following 24-h hypoxia). Increased trimethylation of H3K4 (1.7-fold) and H3K27 (2.2-fold) was detectable within 8 h of hypoxia and was sustained until 24-h hypoxia (Fig. 2a). Conversely, upon restoration of oxygenation, global histone H3K4 and K27 trimethylation returned to baseline at approximately 12–24 h after reoxygenation (Fig. 2a; Additional file 1: Fig. S1A). Similar H3K27me3 dynamics were observed in an independent cancer cell line (Additional file 1: Fig. S1A). Altered H3K9/K14 acetylation status is consistent with an earlier report [39], yet appears to be cell context specific (Additional file 1: Fig. S1A).

To test the dependency of KMD6B activity on oxygen, we measured H3K27me3-directed demethylase activity in vitro as a function of oxygen concentration using purified recombinant KDM6B protein. H3K27 tri-demethylase activity sharply declined from 100% at ambient oxygen levels to 20% activity at 5% oxygen (Fig. 2b). A near linear correlation between oxygen level and enzymatic activity was observed within a physiological range of oxygen pressure (5–1–0.2%). No KDM activity was observed below 0.2% oxygen.

To determine whether the increased H3K4me3 and H3K27me3 levels during hypoxia could be explained by changes in expression of corresponding KMTs, we determined mRNA and protein expression levels of a number of these epigenetic regulators in the context of hypoxia/reoxygenation. Overall, mRNA levels of most confirmed and putative H3K4- and/or H3K27-directed HMTs were low and not substantially affected by altered oxygen pressure within the time frames examined (Additional file 1: Fig. S1B). Correspondingly, protein levels of MLL1/KMT2A, MLL4/KMT2B and SETD1A/KMT2F did not change appreciably in response to oxygen deprivation (Additional file 1: Fig. S1D). Expression of the H3K27me3 HMT-encoding gene *EZH2* decreased in response to oxygen deprivation (Additional file 1: Fig. S1B). In contrast, expression of a number of relevant KDM encoding genes, including *KDM5B* and *KDM6B*, showed a slight increase in hypoxic cells (Additional file 1: Fig. S1C). Protein levels of EZH2 and KDM6B in MCF7 cells followed the mRNA expression profile throughout the experiment. EZH2 protein levels decreased under hypoxia, whereas KDM6B protein levels remained unchanged under hypoxic conditions and dropped upon reoxygenation (Additional file 1: Fig. S1D). Hence, the change in expression levels for both the writer and eraser for the H3K27me3 could not account for the observed global increases in H3K27me3 marks. To explore the possibility that EZH2 activity contributes to increased H3K27me3, MCF7 cells were exposed to hypoxia in the presence or absence of pharmacological inhibitors of EZH2 (EZHi). Reduced normoxic H3K27me3 levels confirmed pharmacological inhibition (Additional file 1: Fig. S1E). Relevantly, however, H3K27me3 levels remain reproducibly higher under hypoxic conditions in the presence of EZHi, compared to normoxic/EZHi conditions. These data are consistent with the notion that global H3K27me3 increases under hypoxic condition despite EZH2 inhibition (Additional file 1: Fig. S1E). Taken together, these findings suggest that physiological levels of hypoxia, including those commonly found in solid tumors, negatively affect histone demethylation activity.

### Selective acquisition of histone methylation at genic regions

We next determined whether changes in oxygen regulate epigenetic states at specific genetic sites. To this end ChIP-seq analysis was performed for both H3K4me3 and H3K27me3 in normoxic (*t* = 0), hypoxic (*t* = 8 and 24 h) and reoxygenated cells (*t* = +8 h). ChIP-seq analysis confirmed enhanced global trimethylation of H3K4 and H3K27 in response to hypoxia, consistent with the immunoblotting findings. The total number of H3K4me3- and H3K27me3-marked genomic regions, from here on referred to as *peaks*, increased 2.6- and 1.2-fold, respectively, at 24-h hypoxia (Fig. 2c, d, upper panels; Additional file 1: Fig. S2A, B). Representative genome tracks illustrate examples of genes that displayed hypoxic gain of H3K4me3 or H3K27me3 levels in response to reduced oxygenation (Fig. 2c, d, lower panels). Bioinformatic calling of enriched regions was validated by ChIP-PCR for a number of representative epigenetic profiles (e.g., H3K4me3- or H3K27me3-only marking, H3K4me3 or H3K27me3 gain; Additional file 1: Fig. S2C). The same data set was used to show that both marks had quantitatively increased at all decorated gene regions (*data not shown*) [43]. These findings show that hypoxia causes an overall reversible epigenome-wide increase of H3K4me3 and H3K27me3, and establish that oxygen is required for constitutive activity of KDM and maintenance of the basal epigenetic state of a large number of genomic regions.

To further enhance comparative epigenomic, enriched sequences were segregated into genic (i.e., “genes” ±5000 bp) and intergenic regions. Under normoxic conditions, trimethylation was highly associated with genic regions: more than 84% of all H3K4me3 peaks located to genes, compared to 41% for H3K27me3 peaks (Fig. 2c, d). Intergenic H3K4me3 association showed a relative increase as a result of acute oxygen deprivation (16% at *t* = 0 to 34% at *t* = 8 h; Additional file 1: Fig. S3A), while intergenic H3K27me3 association did not change significantly (59% at *t* = 0 to 55% at *t* = 8 h; Additional file 1: Fig. S3B).

**Fig. 3:**
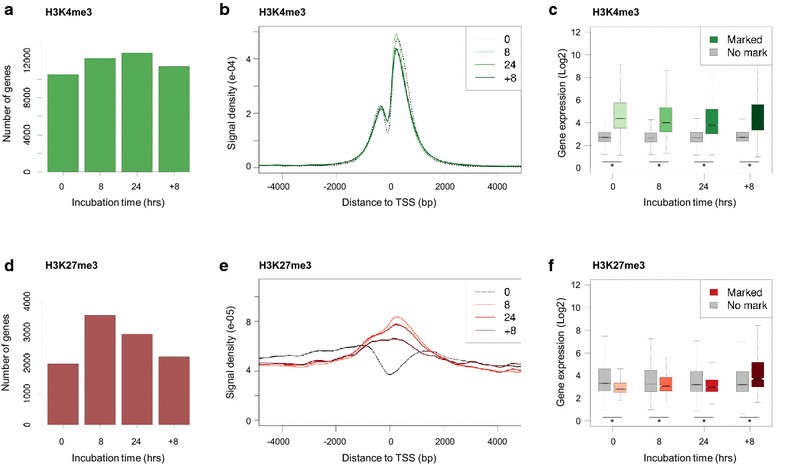
Histone methylation profiles at genic regions during hypoxia. **a** *Bar graph* depicting numbers of H3K4me3-associated genes under indicated conditions (normoxia: 0 h, acute hypoxia: 8 h; chronic hypoxia: 24 h; reoxygenation: +8 h); **b** H3K4me3 peak intensity density distribution proximal to the TSS in relation to oxygen deprivation and reoxygenation; **c** *Boxplots* representing median gene expression in relation to H3K4me3 marking. **d** *Bar graph* depicting numbers of H3K27me3-associated genes; **e** H3K27me3 distribution proximal to the TSS in relation to oxygen deprivation and reoxygenation; **f** *Boxplots* representing median gene expression in relation to H3K27me3 marking. Data presented reflect indicated conditions (normoxia: 0 h, acute hypoxia: 8 h; chronic hypoxia: 24 h; reoxygenation: +8 h). *Asterisks* (**c**, **f**) indicate statistically significant differences (*p* < 0.05)

In normoxic cells, approximately 46% of all genes (*n* = 10,530/22,732) carried H3K4me3 marks, whereas only 9% carried H3K27me3 marks (*n* = 1968) (Fig. 3a, d). The number of H3K4me3-marked genes increased by approximately 16 and 20% after 8 and 24 h of hypoxia, respectively (Fig. 3a). The relative change of H3K27me3 was significantly higher with increases of ±80 and 50% at 8 and 24 h of hypoxia, respectively (Fig. 3d). To profile the hypoxia-induced enrichment of H3K4me3 and H3K27me3 within genic regions, enrichment data were visualized using transcription start site (TSS)-centered plots. Under normoxic conditions, H3K4me3 was prominently enriched around the TSS with a distinctive depletion directly over the TSS; this finding is consistent with earlier reports showing reduced nucleosome presence at this site (Fig. 3b) [44]. In sharp contrast, H3K27me3 marking at normoxia coincided mainly within gene body enrichment, consistent with the reported “blanketing” enrichment of H3K27me3 (Fig. 3e) [43, 45, 46]. Of note, as nearly half of all genes were H3K4me3-enriched already under normoxic conditions (cf. Fig. 3b) and H3K27me3 enrichment was substantially increased (cf. Fig. 3e), hypoxia-induced global H3K27 trimethylation enhanced the frequency of bivalently marked genes (see below).

Reoxygenation nearly fully restored the intergenic–genic ratio of the H3K4me3 peak distribution to that initially observed at ambient oxygen levels (16% at *t* = 0; 18% at *t* = +8; Fig. 2c; Additional file 1: Fig. S3A). Genic enrichment of H3K27me3 was relatively sustained (41 vs. 53%; Fig. 2d; Additional file 1: Fig. S3B). The TSS-centered H3K4me3-enrichment profile did not significantly change as a result of hypoxia or reoxygenation (Fig. 3b), whereas selectively enhanced H3K27me3 enrichment over and around the TSS was partially retained during reoxygenation (Fig. 3e). Combined, the in vitro and in vivo findings support increased overall histone trimethylation at H3K4 and H3K27 under hypoxia and suggest that H3K27me3 marking showed the relatively highest dynamic change under hypoxic conditions.

### Normoxic H3K4me3 and H3K27me3 marking dictate gene activity under hypoxia

To correlate H3K4 and H3K27 trimethylation to gene activity, total RNA samples were collected for analysis at all time points in parallel and ChIP-seq data were compared to expression data before, during and after hypoxic exposure. Gene Ontology (GO) classification confirmed regulation of expected processes in response to hypoxia (Additional file 2: Table S1), consistent with published data [38]. H3K4me3-marked genes were significantly higher expressed at all individual time points (Fig. 3c), whereas H3K27me3-marked genes showed substantially lower median gene transcript levels compared to non-marked genes (Fig. 3f). Relevantly, within a subset of previously reported HIF1A target genes [47], 97.5% showed increased transcriptional activity (Additional file 2: Fig. S4A). Relevantly, the number of H3K4me3-marked HIF1A target genes was increased in hypoxic MCF7 cells (Additional file 1: Fig. S4B). Thus, consistent with expectation, oxygen deprivation induced HIF1A-dependent transcription. To examine whether hypoxia-induced HIF1A regulation directly affected global H3K4me3, H3K4me3 levels were also measured in the absence of HIF1A. This analysis showed that global H3K4me3 levels increase under hypoxic conditions, irrespective of the presence or absence of HIF1A (Additional file 1: Fig. S4C). Thus, HIF1A-mediated stress responses do not significantly contribute to global enhancement of H3K4me3 marking under these conditions.

**Fig. 4:**
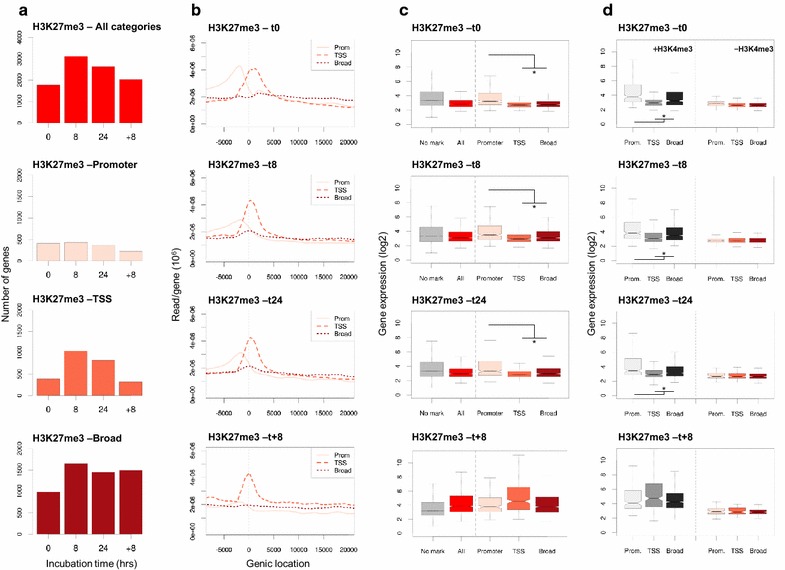
Transcription status is primarily controlled by H3K4me3. **a** *Bar graphs* depicting numbers of H3K27me3-marked genes at indicated experimental conditions (normoxia: 0 h, acute hypoxia: 8 h; chronic hypoxia: 24 h; reoxygenation: +8 h): H3K27me3-enriched loci were classified as (*upper* to *lower panels*): all H3K27me3-enriched genes (*All categories*), promoter (*Promoter*)-, transcription start site (*TSS*)- and gene body (*Broad*)-marked genes; **b** TSS-associated enrichment profiles (average number of reads) for the corresponding H3K27me3 classes (*colored lines*; *solid*: *promoter*, *dashed*: *TSS*, *dotted*: *broad profile*) represent all H3K27me3-marked genes. **c** *Boxplots*: H3K27me3 enrichment was plotted against median expression levels. Comparative analysis was done at (*upper* to *lower panels*) *t* = 0, *t* = 8, *t* = 24 h hypoxia and after reoxygenation (*t* = +8 h). *Subscripts* correspond to non-marked (*no mark*), all H3K27me3-marked genes (*all*), and to promoter, TSS and broad H3K27me3 profiles as indicated. **d** *Boxplots*: H3K4me3 + H3K27me3 and H3K27me3 enrichment only were plotted against median expression levels. Comparative analysis was done at (*upper* to *lower panels*) *t* = 0, *t* = 8, *t* = 24 h hypoxia and after reoxygenation (*t* = +8 h). *Subscripts* correspond to non-marked (*no mark*), all H3K27me3-marked genes (*all*), and to promoter, TSS and broad H3K27me3 profiles as indicated; gene numbers are indicated in *insets*. *Asterisks* (**c**, **d**) indicate statistically significant differences (*p* < 0.05)

We next aimed to correlate the specific intragenic location of H3K27me3 marks to gene expression and determined the effect of gain or loss of these trimethyl marks on transcriptional regulation at all time points. Overall, more gene transcripts were down-regulated than up-regulated in response to hypoxia: Of all expressed genes approximately 58–59% showed reduced transcript levels (Additional file 1: Fig. S5A–C). We then assigned genes to one of three previously defined H3K27me3-enrichment profiles at all experimental time points [48]: (1) a distinct H3K27me3 peak upstream of the TSS (*promoter profile*; −3000/−100 bp with respect to TSS), (2) a distinct peak at the TSS (*TSS profile*; −100/+1000 bp), and (3) no single peak, but instead a “blanketed” distribution over the gene body typical of Polycomb-mediated repression (*broad profile*; +1000/last exon). In control (i.e., normoxic) samples more than half of all H3K27me3-marked genes displayed the broad enrichment profile, whereas the TSS and promoter profiles were equally represented (Fig. 4a, cf. Additional file 1: Fig. S3B). Hypoxia predominantly increased TSS-directed H3K27me3 marking; this relative distribution was maintained for the duration of the hypoxic condition (Fig. 4a, b). Gene expression measurements were plotted for each individual profile and compared to the overall expression of all H3K27me3-enriched genes and non-marked genes. Distinctive TSS marking and gene body enrichment consistently correlated with low gene expression/repression under normoxic and hypoxic conditions (Fig. 4c). In contrast, promoter H3K27me3 enrichment was associated with a significantly higher median gene expression level compared to the total H3K27me3-marked gene population, under both normoxic and hypoxic conditions, and equaled or exceeded expression levels of non-marked genes. H3K4me3 enrichment at H3K27me3-marked genes correlated with a higher median expression level, at normoxic or hypoxic conditions (Fig. 4d; gray boxplots). This effect was strikingly evident for promoter-marked and to a lesser degree for broad H3K27me3-marked loci. In contrast, the presence of H3K4me3 at H3K27me3 TSS-marked genes had relatively little effect on median gene expression levels (Fig. 4d). Thus, TSS-directed H3K27me3 marking correlates with relative transcriptional repression, independent of H3K4me3 status. In contrast, the additional presence of H3K4me3, particularly at promoter-marked and broad H3K27me3-marked loci, correlates with higher gene transcription, irrespective of oxygenation status.

To determine the consequences of hypoxia-induced epigenetic modulation on transcriptional status, loci were first categorized based on their enrichment profile under normoxic conditions (i.e., H3K4me3-only, H3K27me3-only, double-marked or non-marked; Additional file 1: Fig. S6A;a–d). Out of all genes that were single H3K4me3-marked under normoxic conditions, 90% showed persistent H3K4me3 marking and remained transcriptionally active well into hypoxia (*t* = 8, 24 h; Additional file 1: Figs. S6B;a and S6C;a, respectively). H3K4me3-marked genes that gained H3K27me3 marking maintained their median expression level (“H3K4me3 and H3K27me3”; *t* = 8, 24 h hypoxia; Additional file 1: Fig. S6B–C;a). Loss of H3K4me3 (i.e., conversion to H3K27me3-only or non-marked at *t* = 8, 24 h) occurred at relatively few loci; activity status of such loci was low already at *t* = 0 (Additional file 1: Fig. S6B–C;a). Median expression at H3K27me3 single-marked loci was consistently lower compared to loci in the three other categories, independent of whether the H3K27me3 mark was already present at normoxic conditions (Additional file 1: Fig. S6B–C;b) or it was acquired upon oxygen deprivation (Additional file 1: Fig. S6B–C;a,d). H3K27me3-only marked genes (at *t* = 0) showed a relatively strong shift toward gain of H3K4me3 under hypoxic conditions (±30–50%; Additional file 1: Fig. S6A–C;b). Genes that carried both trimethyl marks at *t* = 0 showed an intermediate median expression level compared to H3K4me3-enriched (high expression) and H3K27me3-marked or non-marked genes (low/no expression) (Additional file 1: Fig. S6B;c). Approximately 15% of double-marked genes lost either H3K4me3 or H3K27me3 enrichment in response to acute oxygen deprivation; loss of either trimethyl mark did not affect median expression levels (*n* = 123/*t* = 8 and 151/*t* = 24, respectively; Additional file 1: Fig. S6B–C;c). Of all non-trimethyl-marked genes at *t* = 0, the majority (>80%) retained this epigenetic status until 24 h of hypoxia (Additional file 1: Fig. S6A–B;d). Finally, acquisition of either single H3K4me3 or combined trimethyl marks did not affect median transcription activity at originally H3K27me3 single- or non-marked loci, respectively (Additional file 1: Fig. S6B–C;b,d).

Although the repression-associated TSS H3K27me3 peak intensity distribution was partially maintained upon reoxygenation (cf. Fig. 3e), the correlation between TSS-H3K27me3 marking and transcriptional repression at H3K4me3 and H3K27me3 double-marked loci was lost (Fig. 4c, d; Additional file 1: Fig. S6D;c). Comparable dynamics applied to broad H3K27me3-marked loci. The increased median expression in response to acute reoxygenation (cf. Fig. 3f) suggested (temporary) functional uncoupling of epigenetic marking and transcriptional regulation. In line with this notion, GO classification indicated that numerous cellular processes, including some with no direct relevance to MCF7/breast epithelium biology, were affected by reoxygenation (Additional file 2: Table S3).

Taken together, comparative analysis of altered intragenic epigenetic marking versus gene activity shows that H3K4me3 status is the main determinant of transcriptional activity, under both oxygenated and oxygen-deprived conditions.

### Hypoxia-induced epigenetic bivalency overlaps with bivalent genes in ES cells

Based solely on co-occurrence of H3K4me3 and H3K27me3 marks within genic regions, more than 800 genes were classified as bivalently marked under normoxic conditions; the number of double-marked genes had increased threefold (821 genes, *t* = 0–2200, *t* = 24 h) as a result of hypoxia. To obtain proof that H3K4me3 and H3K27me3 occur within the same allele and are not merely a reflection of different cell pools, bivalent marking was confirmed by sequential ChIP (i.e., ChIP/re-ChIP) at a number of loci (Additional file 1: Fig. S7).

**Fig. 5:**
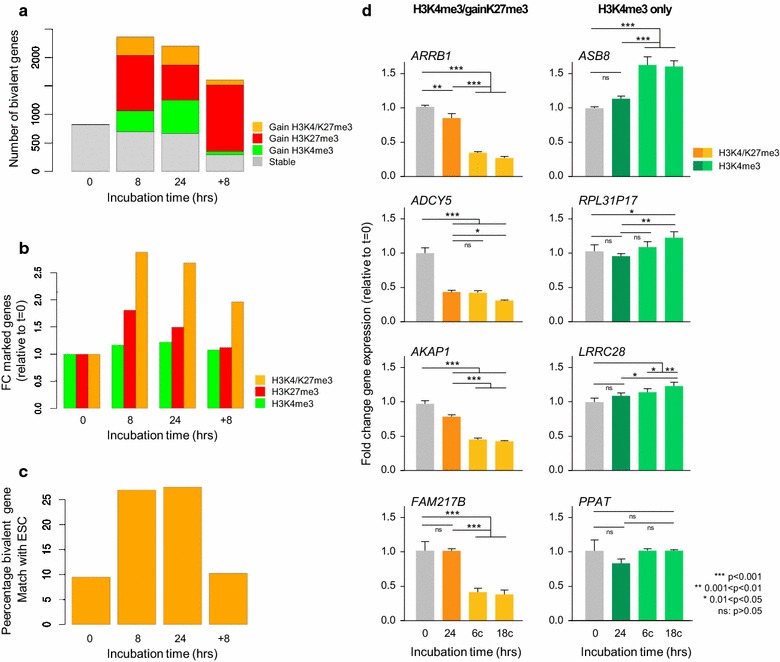
Hypoxia increases genic bivalent marking. **a** *Bar diagrams* depicting increased bivalency (gene numbers) by gain of H3K4me3 (*green*), gain of H3K27me3 (*red*) or gain of both H3K4me3/H3k27me3 marks (*orange*) during hypoxia and reoxygenation. *Gray color bars* indicate the number of bivalent marked genes already present at the indicated time points. **b** *Bar graphs* showing relative changes of H3K4me3-mono, H3K27me3-mono and bivalent marking at the indicated time point. **c** *Bar graph* depicting percentage match of bivalently marked genes in MCF7 cells (at different experimental time points) and in ESC. Comparison of bivalently marked MCF7 loci with published data sets revealed 11–13% (reference data set: [28]; *data not shown*) to 25% target overlap (reference data set: [49]). **d** *Bar graph* depicting changes in expression levels of bivalently marked (4 genes: *ARRB1*, *ADCY5*, *AKAP1*, *FAM217B*; representing a total of *n* = 225) and H3K4me3-only marked (4 genes: *ASB8*, *RPL31P17*, *LRRC28*, *PPAT*; representing a total of *n* = 5755) genes under the indicated experimental conditions. Gene categories were defined based on low expression levels at *t* = 0 (log_2_-based expression value 3.68–6.64). Normoxic expression values (*t* = 0; *left*-*most*, *gray bars*) were set to 1; *orange bars* represent transcriptional activity at the indicated H3K4me3/gain H3k27me3 markers at 24-h chronic hypoxia (24), 6 (6c) or 18 (18c) cycles of oxygen deprivation/reoxygenation; *green bars* represent transcriptional activity at the indicated H3K4me3-only marked loci (24 h, 6c, 18c). Marker expression was normalized to the geo-mean of *beta-actin* and *cyclophilin A. Asterisks* indicate statistical significant differences as depicted in the figure; *ns* nonsignificant difference

The original epigenetic status of loci that had acquired bivalent marking (at *t* = 8 h hypoxia) was traced back to that under normoxic conditions and displayed as a function of experimental oxygenation condition. More than half (55%, 1297 genes) of bivalently marked genes had acquired H3K27me3 alone or in combination with H3K4me3 at 8 h (Fig. 5a, b). Acquisition of bivalency by gain of H3K4me3 under hypoxic conditions revealed a bias toward TSS H3K27me3-marked loci: Of all TSS/H3K27me3-marked genes, 36% was already bivalently marked at *t* = 0; the number of bivalently TSS-marked loci increased ±5.3-fold (at *t* = 8) (Additional file 1: Fig. S8A). Gene body (broad) H3K27me3-enriched genes showed a relatively lower H3K4me3-gain compared to the TSS profile (38% H3K4me3-marked at *t* = 0; ±2.9-fold increase at *t* = 8). Promoter/H3K27me3-marked loci appeared underrepresented with regard to acquisition of bivalency: Due to preexistent TSS/H3K4me3 marking at many loci, the relative increase of bivalent promoter-marked genes was comparatively small under hypoxic conditions (62% of promoter-H3K27me3 genes were H3K4me3-marked at *t* = 0, ±1.3-fold change in gene numbers at *t* = 8). Thus, increased bivalent marking under hypoxic conditions is mainly the result of gain of H3K27me3.

Although bivalency was lost at a substantial percentage of loci upon reoxygenation (68% compared to *t* = 24 h hypoxia), bivalent epigenetic marking persisted at more than 1600 loci (approximately twofold higher compared to normoxia), whereas gain of a single mark was lost upon reoxygenation (Fig. 5b). The stability of acquired bivalency suggests that bivalent H3 trimethylation is relatively resistant to KDM activity at these loci.

Bivalently marked loci are often found among key developmental control genes in (embryonic) stem/progenitor cells [26, 28, 29, 36, 37]. We tested whether the hypoxia-induced bivalently marked gene set in MCF7 cancer cells showed overlap with bivalent genes previously identified in ESC. Approximately 12–25% of the 1500–1700 hypoxia-induced bivalent genes in MCF7 cells overlapped previously identified trimethylated genes in ESC [28, 49]; this match between bivalently marked genes in MCF7 and ES cells increased under hypoxia (Fig. 5c) and was, as described above, relatively sustained under reoxygenation (cf. Additional file 1: Fig. S8A). Comparative GO analysis of both bivalent gene pools revealed a distinctive overlap of functional pathways involved and confirmed the presence of bivalently marked developmental genes; in addition, a number of GO terms (i.a. anti-apoptosis, cell migration, angiogenesis) suggested relevant processes in the context of progressive malignancy (Additional file 2: Table S3). Many of these genes were already bivalently marked at the 8-h hypoxia time point, suggesting that these loci are preset targets for bivalent marking under low oxygen. Hence, our findings suggest that oxygen deprivation in breast cancer cells induces bivalent epigenetic marking at genes which are also bivalently marked in ESC and which control key processes during development.

Transcriptional activity at bivalently marked loci in ESC is known to be low [26, 28, 29, 36]. To determine the consequences of acquired bivalent marking for transcriptional activity, specifically in the context of reoxygenation, we retrospectively traced the expression status of a set of loci that gained bivalent marking (H3K4me3/gain-H3K27me3) during hypoxia and had retained this epigenetic status upon reoxygenation. As more than 85% of these genes showed a relatively low expression level at *t* = 0 (i.e., between 3.68 and 6.64 log_2_-based expression values), we compared these genes to a set of stable H3K4me3-only marked genes that had a comparably low median expression level at *t* = 0, but remained H3K27me3-free under hypoxic conditions. H3K4me3-only marked genes showed a clear and significant progressive reduction of median transcriptional activity under hypoxia and a nearly complete restoration to original (*t* = 0) median expression level upon reoxygenation (Additional file 1: Fig. S8B). In contrast, transcriptional activity at H3K4me3/gain H3K27me3 bivalently marked loci did not significantly change between time points, but instead showed a trend toward a steadily reduced median expression which was maintained upon reoxygenation (Additional file 1: Fig. S8B). This observation suggested that a subset of genes that acquires and retains a bivalent epigenetic state, has selectively maintained responsiveness to transcriptional regulation by H3K27 trimethylation. To test the idea that repeated cycles of hypoxia/reoxygenation induce a progressive reduction of transcriptional activity at these loci, cells were exposed to either 6 or 18 relatively short cycles of hypoxia/reoxygenation, as they would occur in tumors [2, 41]. Expression of 5 randomly chosen genes representing each of both categories (i.e., low expression and *t* = 0, and H3K4me3-only or H3K4me3/gain-H3K27me3) was analyzed by rtPCR. Relevantly, 4 out of 5 bivalent markers showed a significant reduction of transcriptional activity (1 marker showed no significant change; data not shown), whereas H3K4me3-only marked loci showed either increased (3/5) or unchanged (2/5; one marker not shown) expression levels compared to *t* = 0 (Fig. 5d).

These collective findings suggests that oxygen deprivation induces epigenetic bivalency in MCF7 cancer cells, which correlates best with a TSS-specific increase of H3K27me3 at H3K4me3 premarked (i.e., at *t* = 0) genes, and that this acquired epigenetic status is selectively maintained upon reoxygenation despite global loss of trimethylation. In addition, the data suggest that acquired bivalent marking is relatively stable and prevents transcriptional variability during hypoxia and reoxygenation at a select set of genes that overlap with bivalently marked genes in ESC.

## Discussion

Here, we report that hypoxia, through enzymatic inhibition of KDMs, increases histone H3 trimethylation in cultured MCF7 breast cancer cells and in breast cancer xenografts. Although most changes in H3K4me3 and/or H3K27me3 levels are transient and return to baseline levels upon reoxygenation, we provide evidence that some H3K27me3 enrichment persists at defined genomic regions. During hypoxia increased histone trimethylation leads to acquisition of bivalent marking at numerous loci and transcriptional activity at these loci is stably maintained at a low level with a trend toward reduced expression relative to H3K4me3-only marked loci. As a subset of these bivalently marked genes represents previously identified bivalently marked genes in ESC, these findings suggest a link to tumor cell plasticity and/or stemness.

### Hypoxia-induced reversible histone trimethylation

Comprehensive insight into transcriptional reprogramming in response to changing microenvironments requires systematical genome-wide mapping of epigenetic marks as a function of cell type, differential state and microenvironment [24]. We here studied dynamic changes in histone trimethylation in the context of cellular adaptation to changes in oxygenation status (hypoxic stress, reoxygenation). We were able to reproduce the reported correlation between gene body-associated “blanketing” H3K27me3 profile and transcriptional repression, thereby validating our analytical approach [43]. The prominent TSS-centered H3K4me3 enrichment at expressed loci and the marked low nucleosome abundance right over the TSS are also consistent with earlier findings [27].

Increased global histone trimethylation during hypoxia could theoretically result from increased histone methyl transferase activity or reduced histone demethylase activity. In line with the original hypothesis that 2-OGDO family proteins to which the Jumonji class of KDMs belong, depend on molecular oxygen for their activity, we provide direct evidence that KDM6B activity is inhibited under hypoxic conditions. The steep decline of global H3K4me3 and H3K27me3 levels following reoxygenation is likely to be due to increased KDM protein expression during the course of hypoxic treatment and rapid KDM activation by molecular oxygen. By inference, inhibition of KDMs in the absence of molecular oxygen is likely to be responsible for the increased TSS-directed bivalency. This finding implies that maintenance of the epigenetic status at these genes under basal (i.e., normoxic) conditions likely requires continuous dynamic remodeling by KDMs, among other enzymes, and that such loci may be uniquely sensitive to specific signals from the microenvironment (e.g., cofactor availability). Although the involvement of demethylases was recently addressed in the context of hypoxia-induced increased H3K4me3 marking [50], our studies provide additional insight into the effects of reoxygenation on epigenomic and transcriptomic responses. Our combined observations thus establish that oxygen sensing by KDM represents a direct link between the microenvironment and epigenetic regulatory mechanisms.

### Hypoxia-induced epigenetic changes versus gene expression

Our comparative analysis of ChIP-seq and expression array data sets revealed a clear positive correlation between TSS-directed H3K4me3 enrichment and gene expression under all conditions. H3K27me3 displayed multiple distinct genic enrichment profiles, in line with previous publications [48, 51]. H3K27me3 enrichment across the gene body (*broad* profile) followed the classical Polycomb-associated repressive H3K27me3-enrichment profile, which is referred to as “blanketing” [45, 46]. Thus, the strong promoter/TSS association of genic H3K4 trimethyl marking versus the clear gene body-directed genic H3K27me3 marking demonstrate that genomic decoration with H3K4me3 and H3K27me3 is consistent with their known distribution and roles in gene regulation. In addition, the selective changes in genic marking indicate that increased H3 trimethylation during hypoxia is not occurring in a random fashion.

Sustained H3K4me3 marking upon hypoxic treatment appears epigenetically dominant over genic H3K27me3 acquisition in regard to transcriptional regulation. Although a possible involvement of altered RNA stability can formally not be excluded, transcriptional changes should in principle be detectable over the time intervals in our experimental setting (e.g., gain of H3K4me3 showed a positive trend with increased expression). The exact relevance of Polycomb repressive complex-associated (PRC) H3K27me3 marking and enrichment at different intragenic positions is currently not fully understood. The H3K27me3 mark is bound by both PRC1 and PRC2 proteins; these interactions are likely important for reestablishment and thus transfer of an epigenetic state [52–57]. H3K27me3-dependent target-gene silencing can act via distant enhancer-looping to gene promoters in insect and in mammalian systems [58]. However, PRC proteins are known to localize at active RNA polymerase 2 (POLR2) promoters in *Drosophila* and to interact with basal transcription factors (TF) [59]. In keeping with this notion, we recently showed that transcriptional activation at a defined PRC1-target locus did not require loss of H3K27me3 marking, but PCR1/chromatin dissociation [60]. In addition, PRC associates with splicing factors, revealing an as of yet poorly understood additional role in regulation of gene expression [61]. Hence, our observations herein suggest that, once marked for transcription, acquisition of genic H3K27me3 marking may not be sufficient for transcriptional repression *per se*, but is likely to depend on additional (epigenetic) events.

### Implications of increased bivalency under hypoxia

We show that a subset of H3K4me3-marked genes acquired bivalency principally by gain of TSS-directed H3K27me3 marking relative to promoter and gene body marking; this resulted in bivalent marking of a substantial number of genes (±950–1100; at *t* = 8 and 24 combined). Despite the global demethylation following reoxygenation, bivalent marking persisted at a substantial number of loci 8 h into restoration of oxygen levels. Co-occurrence of both methyl marks was first described in embryonic stem cells and is thought to mark key developmental control genes as “poised” for transcriptional activation. Specific H3K27me3 enrichment at promoter regions, in combination with a marked depletion of gene body-directed H3K27me3 marking, overlaps with H3K4me3-enrichment profiles and appears permissive for active transcription; such bivalently marked genes are typically expressed at low levels in stem cells [26, 28, 29, 36, 37, 45]. Interestingly, the bivalent gene subset in hypoxic MCF7 cells represents a substantial number of genes previously identified as bivalently marked genes in ESC.

We observed that within the bivalently marked subset, TSS-directed H3K27me3 acquisition with preexistent H3K4me3 marking dampened transcriptional fluctuation of the corresponding genes compared to H3K4me3-only marked genes. Relevantly, upon repeated exposure to hypoxia, as would occur in tumor cells in situ [2], transcriptional activity was significantly and specifically reduced at these bivalent loci. This finding strongly supports the notion that transcriptional activity at these loci is specifically controlled by H3K27me3 demethylases and that such loci may also be sensitive detectors of oxygen and other KDM cofactor availability. Of relevance, KMTs (e.g., for H3K4me3) are often found in close conjunction with KDMs for functionally opposing epigenetic marks (e.g., H3K27me3); together these paired epigenetic modulators are thought to reinforce transcriptional decisions, a concept termed: *co*-*stability* [62]. It is tempting to speculate about a similar role for cooperativity between H3K4me3 KMTs and H3K27me3 KDMs in the establishment of bivalency under KDM-inhibitory conditions, as our findings identify a subset of genes which is apparently preset for acquisition of bivalent marking; transcriptional activity at these loci is likely to be specifically controlled by H3K27me3 KDM. It is currently unclear whether and how these loci are protected from KDM activity (and/or KMTs) upon reoxygenation, although it is conceivable that some other local epigenetic aspect (e.g., inhibitor recruitment or higher-order chromatin structure) mediates sustained methylation status. Irrespective of the exact underlying mechanism, our findings identify the H3K27me3 mark as most prone to dynamic change in response to hypoxia/reoxygenation and define a novel role for oxygen in epigenetic regulation of stress responses.

### Hypoxia, epigenetics and cancer

Although the relevance of reduced gene expression at any of the select bivalent loci in regard to (tumor) stemness is currently unknown, numerous reports support a connection between hypoxia and stem cell biology. Stem cell niches are known to be hypoxic [63–66]. Hypoxia enhances iPSC formation in vitro [8–11]. Repeated oxygen deprivation and reoxygenation are known to promote tumor stem cell properties, metastasis, and patient prognosis and adaptive responses to hypoxia severely decrease efficacy of both ionizing radiation and chemotherapy [3–5]. We have recently shown that oxygen withdrawal results in the acquisition of stem cell phenotypes in human mammary epithelial cells via inhibition of oxygen-dependent H3K27me3 demethylases KDM6A/B and consequential reduction of DICER expression and derepression of the miR-200 target ZEB1 [40]. Our combined findings suggests that whereas increased H3K27me3 decoration under hypoxic conditions is an epigenome-wide event, it is likely not sufficient for acquisition of stemness like properties, as sphere-forming capacity appears to be a stochastic event [40]. As cancer development is thought to be sustained by cancer stem cells [67–69], it is tempting to speculate about a possible role for bivalent marking in re-establishing a “poised” gene status, and that hypoxic microenvironment selects for or drives tumor plasticity through acquisition of a less differentiated epigenome.

## Conclusions

Our understanding of altered chromatin states and the role of epigenetic modifiers in the context of embryogenesis, spermatogenesis, metabolic disorders and tumorigenesis has progressed rapidly [70, 71]. Recent advances have implicated mutation and/or dysregulated expression of KMTs and KDMs in cancer. Identification of mutations and deregulated expression of canonical histone proteins, histone variants and histone chaperones, in addition to chromatin-associated epigenetic regulatory factors, emphasizes the relevance of protecting genomic as well as epigenomic integrity to prevent malignant tumor development [72, 73]. Whereas knowledge on regulation of KMT expression and/or activity under hypoxic conditions is limited, expression of a number of KDMs is known to be mediated by HIF1A [50, 74–77]. Dysregulated expression of KMTs and KDMs is known to correlate with tumor cell survival, growth and increased malignancy [78–92]. Combined these observations suggest the existence of dynamic feedback mechanisms among these factors that generate favorable tumor survival responses and microenvironmental conditions. Our study adds another level of epigenetic involvement in tumor biology to these observations: local and global chromatin modification in response to changes in the hypoxic tumor microenvironment. This aspect of tumor physiology is likely to affect a substantially larger cohort of patients compared to genetic mutation in epigenetic regulatory pathways.

## Methods

### Immunohistology

All animal experiments were approved by the local ethical committee from (Maastricht University; DEC license 2012-107). The murine invasive lobular breast carcinoma cell line Kep1.11 was injected into the mammary fat path of NMRI-nu mice. When tumors reached an average volume of ±250 mm^3^, animals were injected with pimonidazole (60 mg/kg i.p.) 1 h prior to killing. Tumors were excised and snap-frozen for histological analysis. Consecutive 5-μm sections were air-dried, acetone-fixed and rehydrated using PBS-Tween (0.2%) before blocking (30 min) with normal goat serum (5%). Slides were incubated for 30 min with rabbit anti-pimonidazole (Hypoxyprobe-1, Bioconnect; 1:150), rabbit anti-H3K27me3 (07-499, Millipore; 1:400) or rabbit anti-H3K4me3 (Ab8580, Abcam; 1:400). This was immediately followed by incubation (30 min) with rat anti-mouse CD31 (clone MEC13.3, BD Biosciences; 1:500) in a humidified chamber at 37 °C. After washing in PBS-Tween, sections were incubated with secondary antibodies (goat anti-rabbit IgG Alexa Fluor 594, 1:500 or goat anti-rat IgG Alexa Fluor 488, 1:750; Invitrogen) for 45 min at 37 °C, washed and mounted with fluorescent mounting medium (DakoCytomation). Automated image acquisition was done using Micromanager 1.4 software for [93] and spinning disk confocal microscopy (Olympus BX51WI) equipped with a Hamamatsu EM-CCD C9100 digital camera, a motorized stage (Ludl Mac 2000) and a 10× objective. Image stitching and thresholding was performed using ImageJ v1.49e software.

### Cell culture

MCF7 (human mammary adenocarcinoma) and DU145 (human prostate carcinoma) cells (ATCC) were cultured at 37 °C, 5% CO_2_, 100% humidity in Dulbecco’s modified Eagle medium–nutrient mixture F-12 (DMEM/F12 1:1; MCF7) and DMEM McCoy’s 5A medium (DU145). The culture medium was supplemented with 10% fetal calf serum (FCS), 200 mM l-glutamine and antibiotics. For hypoxic exposure, cells were transferred to a MACS VA500 microaerophilic workstation (Don Whitley Scientific, Shipley, UK) for the indicated duration. The atmosphere in the chamber consisted of <0.02% O_2_, 5% H_2_, 5% CO_2_ and residual N_2_. For reoxygenation, cells were transferred back to the regular tissue culture incubator at ambient oxygen levels (21%). In some experimental conditions cells were exposed to EZH2 inhibitors 5 µM UCN-1999 (source) or 5 µM GSK343 (source); both inhibitors were dissolved in DMSO. Repeated oxygen deprivation/reoxygenation was achieved using equipment and software developed by the Engineering Department (Instruments Development, Engineering and Evaluation) of Maastricht University Medical Centre, as previously described [94]. Briefly, MCF7 cells were seeded in 10-cm dishes (5 × 10^5^ cells/cm^2^) and exposed in triplicate to cycles of normoxia/hypoxia (6 or 18 cycles; 1 h 21% O_2_ alternated by 1 h <0.02% O_2_); cycling was preceded and ended by an 8-h <0.02% O_2_ exposure. The atmosphere in the chambers always contained 5% CO_2_ and residual N_2_. After exposure, cells were processed for RNA isolation as described below.

### Protein isolation and immunoblotting

Cells were grown to approximately 70% confluence before they were transferred to the hypoxic chamber. For protein extraction cells were washed twice with cold PBS and lysed in RIPA buffer (150 mM NaCl, 1% NP-40, 0.5% w/v sodium deoxycholate, 0.1% SDS, 50 mM Tris at pH 8.0, 5 mM EDTA) supplemented with protease and phosphatase inhibitors (5 mM benzamidine, 5 µg/ml antipain, 5 µg/ml leupeptin, 5 µg/µl aprotinin, 1 mM sodium vanadate, 10 mM sodium fluoride, 10 mM pyrophosphate, 10 mM ß-glycerophosphate, 0.5 mM DTT and 1 mM PMSF). Lysates were subjected to two freeze–thaw cycles in liquid nitrogen, followed by sonication on ice with a probe sonicator (Soniprep 150; MSE, London, UK) for 12 cycles (1 s ON, 1 s OFF) with amplitude 5. After 10-min centrifugation at 13.200 rpm (4 °C), the supernatant was transferred to a fresh tube and protein concentration was determined using a BCA protein assay kit (Pierce/Thermo Fisher Scientific, Rockford, IL, USA) according to the manufacturers’ protocols on a Benchmark 550 Micro-plate Reader (Bio-Rad). For immunoblotting (IB) equal amounts of protein were boiled in Laemmli buffer for 5 min and loaded on 9–15% polyacrylamide gels. Following separation by SDS-PAGE, proteins were transferred onto polyvinylidene fluoride (PVDF) membranes (GE Healthcare). Ponceau S (Sigma) staining was used to check protein transfer. Subsequently, PVDF membranes were blocked with 3.4% nonfat dry milk (Protifar; Nutricia, Zoetermeer, the Netherlands) in PBS containing 0.1% Tween 20 (pH 7.5) for 1 h at RT, followed by an overnight incubation at 4 °C with the primary antibody (see Additional file 2: Table S2). Antibodies used include: anti-H3, anti-H3K4me3, anti-JMJD3 and anti-bTubulin (ab1791, Ab8580, ab85392, ab6046; Abcam, Cambridge, UK), H3K27me3 (07-449; Upstate Biotechnology/Millipore, Waltham, MA, USA), anti-EZH2 (courtesy A. Bracken [45]), anti-MLL1, anti-MLL4 and anti-SETD1A (A300-374A, A300-113A, A300-289A; Bethyl Laboratories), anti-HIF1A (Clone 54; BD Bioscience), anti-CA9 (M75; courtesy S. Pastorekova [95]), anti-aTubulin (T6074; Sigma) and b-Actin (C4, 69100; MP Biomedicals, Solon, OH, USA). After extensive washing with PBS/0.2% Tween 20, bound antibodies were visualized with corresponding horseradish peroxidase-conjugated secondary antibodies (1 h at RT): rat anti-mouse (DAKO, Glostrup, Denmark) and donkey-anti-rabbit (Jackson Lab, Bar Harbor, ME, USA) or by using IRDye 800CW or 670RD secondary antibodies (LI-COR). Signals were detected on autoradiograms using enhanced chemoluminescence (ECL; Pierce). Immunoblots were imaged and quantified using Quantity One software (Bio-Rad) or the Odyssey CLx imaging system (LI-COR) and ImageStudio Lite v.5.2.5 (LI-COR). Plots were generated using GraphPad Prism, version 4.03, for Windows (GraphPad Software, San Diego, CA, USA). Data were statistically analyzed by performing two-tailed paired *t* tests using Microsoft Excel. Data given are expressed as mean ± standard deviation (SD) and considered significant at *p* < 0.05. *, ** and *** indicate *p* < 0.05, 0.01 and 0.001, respectively.

### In vitro KDM assay

KDM6B (JMJD3) activity was measured using the JMJD3 Chemiluminescent Assay Kit (Tebu-Bio, The Netherlands) according to the manufacturers’ instructions. Application of 25–100 ng recombinant KDM6B protein produced a dose-dependent response; the assay was performed with 50 ng recombinant protein. Briefly, 8-well strips, precoated with methylated histone H3 peptides, were hydrated and incubated with recombinant KDM6B for 1 h exactly. Upon washing with TBST (50 mM Tris, 150 mM NaCl, 0.05% Tween 20), wells were incubated with blocking buffer for 10 min. This first part of the assay was performed in an atmosphere with controlled oxygen levels using a MACS VA500 microaerophilic workstation (DW Scientific, UK). All solutions and materials were pre-equilibrated under the desired conditions. Incubation with ethylenediaminetetraacetic acid (EDTA, 50 mM) provided a negative control. Detection of demethylated H3 substrate was carried out under atmospheric oxygen concentration. Primary and HRP-labeled secondary antibodies (provided with the kit) were incubated for 1 h and 30 min, respectively. A chemiluminescent signal was measured upon the addition of an HRP substrate.

### Chromatin immunoprecipitation (ChIP) assays

MCF7 cells were transferred to hypoxic culturing conditions for the indicated durations and immediately fixed in phosphate-buffered saline (PBS) containing 1% formaldehyde to avoid reoxygenation. Cross-linking was allowed to proceed for 10 min at room temperature and stopped by a 5-min incubation with glycine at a final concentration of 0.125 M. Fixed cells were washed twice with PBS and harvested in SDS buffer (50 mM Tris at pH 8.1, 0.5% SDS, 100 mM NaCl, 5 mM EDTA), supplemented with protease inhibitors (aprotinin, antipain and leupeptin all at 5 µg/ml and 1 mM PMSF). Cells were pelleted by centrifugation and suspended in IP buffer (100 mM Tris at pH 8.6, 100 mM NaCl, 0.3% SDS, 1.7% Triton X-100, and 5 mM EDTA), containing protease inhibitors. Cells were disrupted by sonication, yielding genomic DNA fragments ranging from 200 to 1000 base pairs, with a bulk size of 200–500 bp. For each immunoprecipitation 10–20 million cells were used. Approximately 1.2 ml of lysate was precleared by adding of 35 µl of pretreated protein A beads; pretreatment: beads were washed six times in IP buffer and incubated in blocking solution (Protein A-Sepharose/CL-4B, GE Healthcare, Piscataway, NJ, USA); 0.5 mg/ml fatty acid-free BSA, Sigma; and 0.2 mg/ml herring sperm DNA in TE (100 mM Tris at pH 8.1; 5 mM EDTA) for 60 min at 4 °C, followed by centrifugation (3000 rpm, 60 s, 4 °C). Aliquots (12 µl) of precleared suspension were put aside as input (1/100th) DNA and kept at 4 °C. ChIPs were performed and analyzed as described previously with minor adjustments [45]. Samples were immunoprecipitated overnight at 4 °C with primary antibodies. Antibodies used include: anti-H3K4me3 (Ab8580; Abcam), anti-H3K27me3 (07-449; Upstate) and anti-HA (sc-805; Santa Cruz Biotechnology, Santa Cruz, CA, USA) as a negative controls. Immune complexes were recovered by adding 40 µl of blocked protein A beads (GE Healthcare) and incubated for 4 h at 4 °C. Beads were washed three times in 1 ml chilled mixed micelle buffer (20 mM Tris at pH 8.1, 150 mM NaCl, 5 mM EDTA, 5% w/v sucrose, 1% Triton X-100 and 0.2% SDS), twice in 1 ml chilled buffer 500 (50 mM HEPES at pH 7.5, 0.1% w/v sodium deoxycholate, 1% Triton X-100 and 1 mM EDTA), twice in 1 ml chilled LiCl detergent wash buffer (10 mM Tris at pH 8.0, 0.5% sodium deoxycholate, 0.5% NP-40, 250 mM LiCl and 1 mM EDTA), and once in 1 ml chilled TE. For sequential-ChIP (ChIP/reChIP), the primary precipitate was incubated for 30 min at 37 °C in 250 µl elution buffer (1% SDS, 0.1 M NaHCO3; under constant agitation) to elute protein/DNA complexes. Supernatants (30-s centrifugation, maximum speed) were diluted in 3.2 ml IP buffer and incubated for 5 min (under constant agitation; 4 °C); 100 µl per original IP sample was saved as input (1/10th); the remainder was split in three 1-ml aliquots and re-incubated with primary antibodies (e.g., anti-H3K4me3, anti-H3K27me3 or control) overnight, as described above. Pretreated protein A beads (20 µl) were added and incubated for 4 h (constant agitation, 4 °C). Beads were collected by centrifugation (3000 rpm, 60 s, 4 °C), washed in consecutive steps in mixed micelle buffer, buffer 500, LiCl detergent wash buffer and TE, as outlined above for the primary precipitates. Immune complexes were eluted for 2 h at 65 °C in 250 µl elution buffer under constant agitation at 1000 rpm, and supernatants were collected (30-s centrifugation, maximum speed). For PCR analysis, 250 µl elution buffer was added to input DNA samples and these were processed in parallel with eluted samples. Cross-links were reversed overnight at 65 °C, followed by a 2-h digestion with RNAse A at 37 °C and 2 h proteinase K (0.2 µg/µl) at 55 °C. DNA fragments were recovered using QIAquick PCR purification columns (Qiagen, Hilden, Germany), according to manufacturers’ instructions. Samples were eluted in 75 µl EB, and the immunoprecipitated DNA was checked for enrichment using real-time PCR and quantified by fluorescence detection using Quant-iT™ Picogreen® dsDNA Reagent (Molecular Probers/Invitrogen, Eugene, OR, USA) before deep sequencing was applied. ChIP- and sequential-ChIP primers are listed in Additional file 2: Table S4.

### Deep sequencing

Input and ChIP samples were further processed and sequenced at the Ontario Institute for Cancer Research using the Illumina next-generation sequencing platform. Processing involved size selection to enrich for mononucleosome-sized fragments, linker annealing and PCR amplification. Each resulting library was subsequently loaded onto individual lanes of a flow cell and sequenced using the 36-bp paired-end protocol on the Illumina Genome Analyser IIx (GAIIx). In order to obtain sufficient sequencing depth additional lanes were sequenced if necessary. All data obtained from each individual sample were pooled.

### Genome alignment, normalization, background correction; identification of enriched regions

Image processing and base calling were performed using Illumina software tools provided by the manufacturer. Subsequent paired-end genome alignment was performed using Novoalign with Human Genome 18 (HG18) used as a reference genome. Only uniquely aligned reads were used for further analysis. To remove PCR artifacts all data were collapsed prior to peak calling. To identify enriched regions in the ChIP samples relative to the input control, the peak caller Findpeaks (version 4.0) was used. For H3K4me3 the default settings were used, whereas for H3K27me3 the settings were adjusted in order to detect blanketing enrichment next to sharply defined peaks [43].

H3K27me3 is known to cover large chromosomal regions instead of sharp defined peaks [45, 46]. H3K27me3 data sets were normalized based on identification of regions with stable H3K27me3 enrichment between all samples analyzed, to enable quantitative comparisons. To this end we developed a standardized protocol to define and summarize H3K27me3 enrichment using deep-sequencing analysis. A detailed description of the protocol and the scripts used will be published elsewhere [43]. Briefly, so-called *invariant regions* were identified by calculating for each 20-Kb window in the genome the relative enrichment for high-intensity H3K27me3 peaks (peak height >90th percentile) using the ACME algorithm; 20-Kb regions enriched in all samples analyzed were defined as invariant, and neighboring invariant regions were merged. The cumulative area under the curve for all peaks within these invariant regions was scaled relative to the smallest value among the samples. After normalization, a single cutoff value for all samples was set at the peak height) above which the H3K27me3 data correlate with the ChIP-seq data of a known H3K27me3-binding protein under normoxic conditions (*t* = 0; data not shown). Peaks below this threshold were considered background noise (i.e., biologically irrelevant). To add further biological validity to this threshold, we defined a set of 1137 genes whose expression levels were above the 95th percentile for all samples analyzed, i.e., consistently highly expressed genes. Any repressive H3K27me3 marks present in these genes can be considered background noise, and indeed, for the vast majority of highly expressed genes H3K27me3 peaks were found to be below our calculated threshold.

### RNA isolation, gene expression microarray processing and analysis

For rtPCR analysis, total RNA was isolated using RNeasy minikit (Qiagen, Hilden, Germany) according to the manufacturers’ protocol. Quantity and quality of the RNA were determined by 260/280 and 260/230 nm absorbance measurements, respectively, using the Nanodrop (Witec AG, Luzern, Switzerland). Total RNA (0.5 µg) for each sample/replicate was converted into first-strand cDNA using the SensiFAST™ cDNA synthesis kit (Bioline, London, UK) according to the manufacturers’ instructions. Gene expression was determined by rtPCR using the LightCycler^®^ 480 (Roche Life Science, Penzberg, Germany) in combination with LightCycler^®^ 480 software (Roche Life Science); rtPCR was performed on 10 ng cDNA using the qPCR SensiMix™ SYBR and Fluorescein kit (Bioline) and 37.5 nM primer in 384-well plates (Roche Life Science). For each primer pair a standard curve was generated with a serial dilution of a cDNA pool; rtPCR data were analyzed according to the LinRegPCR method. All values were normalized to *beta*-*actin* and *cyclophilin A*; the control condition was used as a reference (set to 1). Primer sets for the selected genes were developed with Primer Express version 2.0 (Applied Biosystems, Foster City, CA, USA) using default settings (Additional file 2: Table S5).

RNA for microarray application was isolated using RNeasy minikit (Qiagen, Hilden, Germany) according to manufacturer’s protocol. Isolations were performed in triplicate. Total RNA samples were analyzed using the Affymetrix expression array platform (Affymetrix Gene Chip 1.0 ST). After scanning, data preprocessing and data analysis were done with R (http://www.R-project.org; version 2.15) using Bioconductor (http://www.bioconductor.org; version 2.11). Data were background-corrected using gcRMA [96] and normalized using GRSN rank-invariant normalization [97]. Updated Ensembl-based probe set annotation was used from Brain Array (http://brainarray.mbni.med.umich.edu/brainarray/). The expression level of an individual gene was defined as the average of all probe sets representing this gene on the array. The lower detection threshold of gene expression was set at the maximum expression levels of Y chromosome genes (i.e., non-relevant in the context of female MCF7 cell line; log_2_-based expression value: 3.68). Genes were considered expressed well above background when expression exceeded 100 (non-log_2_ transformed expression units; log_2_-based expression value: ±6.64) for at least one independent time point. Statistical analysis of differential gene expression was performed using two ANOVA models: (1) a model for changes occurring under hypoxia (*t* = 0, *t* = 8 and *t* = 24; *n* = 3 for each time point) and (2) a model for changes occurring under reoxygenation (*t* = 24, *t* = +8 and *t* = 0 as putative endpoint; *n* = 3 for each time point). Acquired *p* values were corrected for multiple testing using the *q* value package in R. Genes were called significantly regulated if genes are expressed (>100 for 1 or more time points), the log_2_-based fold change between 2 independent time points is ≥2 and a corrected *p* value ≤0.05 for either the hypoxia model or reoxygenation model. Genes which were not represented on the microarray were not considered in the analyses.

### Integration of microarray gene expression data and H3K27me3 and H3K4me3 ChIP-seq data

The microarray gene expression data and the H3K27me3 and H3K4me3 enrichment ChIP-seq data were integrated using the latest Ensembl genome annotation (HG18). For the identification of H3K27me3- and/or H3K4me3-enriched genes, we defined a gene as the region between its 5′ (most upstream TSS) and 3′ (last exon) end plus 5-kb regulatory regions up and downstream, respectively. A gene was called enriched or marked when there was at least one ChIP-seq peak above background level present within this region as determined by the enrichment finding procedure. Gene Ontology enrichment analysis was performed using topGO [98, 99]. Comparative GO analysis with embryonal stem (ES) cell bivalent markers was based on published data [28]. Overlap of bivalently marked targets in MCF7 and in ES cells was determined by using the original reported data set of 332 bivalent genes [28] and independently by using a recent metaset of 2550 bivalent genes (consistent across multiple data sets; [49]). The data sets supporting the results of the current study are available in the Gene Expression Omnibus (GEO) repository (http://www.ncbi.nlm.nih.gov/geo/) under accession number GSE71031 (subseries GSE70805 and GSE71030). Temporary reviewer link:

http://www.ncbi.nlm.nih.gov/geo/query/acc.cgi?token=mpizumgavfqrbun&acc=GSE71031.

### Data visualization

All data visualization was carried out in R, including creation of histograms, gene tracks, genome plots, pie charts, TSS plots, boxplots, scatter plots. For the TSS peak intensity plots, all genes were considered in the same orientation (from 5′ to 3′ end), and the average peak intensity was depicted for a region defined in number of base pairs surrounding the TSS, as indicated. Boxplots show the 25th and 75th quartile (as indicated by the box), the median (indicated as a line within the box), and the whiskers indicate the 5 and 95% percentile, respectively. Notches in boxplots indicate 95% confidence intervals of the median. Differences between groups visualized in the boxplots were tested using the nonparametric Wilcoxon signed-rank test, with significant differences (*p* < 0.05) visually indicated in each.

### H3K27me3 region-specific profile analysis

To study region-specific enrichment profiles for H3K27me3, each gene was assigned to the promoter, the TSS or the Broad class, analogous to a previously published analysis [48]. To classify all genes, each gene was first divided into three regions: the promoter region [−3000/−100 base pairs (bp) in relation to the TSS], the TSS region (−100/1000 bp) and the broad region (+1000 bp to the last exon). Genes were allocated to the different classes based on which of the three regions contained the largest amount of summarized enrichment (sum of normalized peak intensities within a region) scaled to the size of each region: Promoter class genes contain 25% more enrichment in the promoter region compared to the TSS and broad regions; TSS class genes contain 25% more enrichment in the TSS region compared to the promoter and broad regions; and lastly, the Broad class genes contain peaks above background level in the broad region and are not TSS and Promoter class. Genes shorter than 4000 bp were excluded from the analysis, as they were too small to reliably assign them to either profile. Genes not meeting the above criteria were not considered for region-specific enrichment profile analysis.

## Additional files

**Additional file 1:** Additional figures.

**Additional file 2:** Additional tables.

## Abbreviations

2-OGDO: 2-oxoglutarate dioxygenases
ChIP-seq: chromatin immunoprecipitation-deep sequencing
DICER: double-stranded RNA-specific endoribonuclease
ESC: embryonic stem cells
EZH: enhancer of zeste human
EZHi: EZH inhibitor
H3K27me3: histone 3 lysine 27 trimethylated
H3K4me3: histone 3 lysine 4 trimethylated
HIF1A: hypoxia-inducible factor 1 alpha
JARID: Jumonji AT-rich interactive domain
KDM6B/JMJD3: lysine demethylase 6B/Jumonji D3
KMD: lysine demethylase
KMT: lysine methyltransferase
PHD: prolyl hydroxylase
POLR2: RNA polymerase 2
PRC: Polycomb repressive complex
pVHL: von Hippel–Lindau tumor suppressor
rtPCR: real-time polymerase chain reaction
TSS: transcription start site
