# Supplementary material for: Hypoxia increases genome-wide bivalent epigenetic marking by specific gain of H3K27me3
Source: Epigenetics Chromatin. 2016 Oct 26;9:46. doi: 10.1186/s13072-016-0086-0 (PMC5080723; doi:10.1186/s13072-016-0086-0)
Supplement: Supplementary file 1 — Additional file 1: Additional figures. [file 13072_2016_86_MOESM1_ESM.pdf]

Figure S1

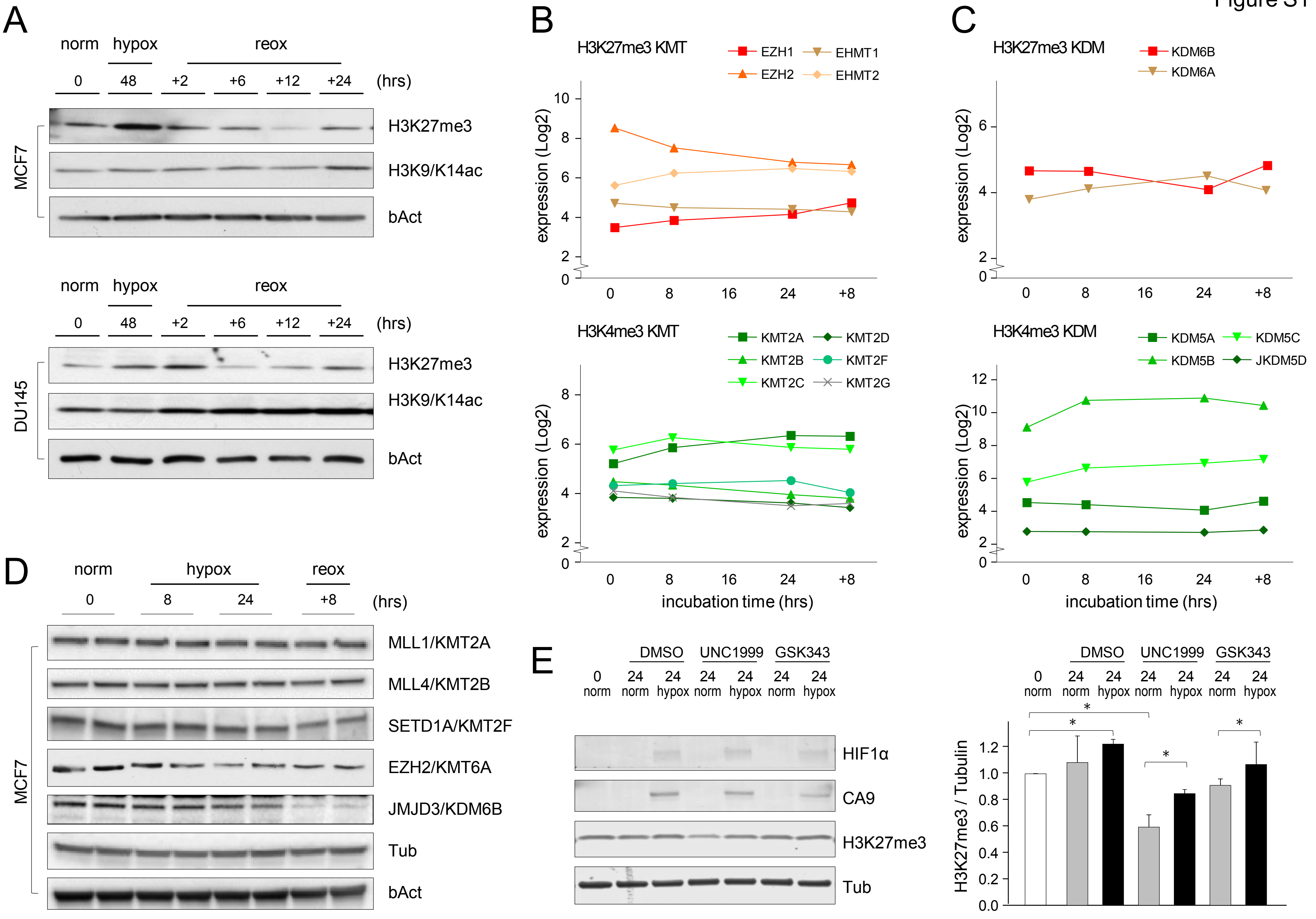

A
norm
hypox
reox
0
48
+2
+6
+12
+24
(hrs)
MCF7
H3K27me3
H3K9/K14ac
bAct
DU145
H3K27me3
H3K9/K14ac
bAct
B
H3K27me3 KMT
EZH1
EZH2
EHMT1
EHMT2
expression (Log2)
H3K4me3 KMT
KMT2A
KMT2B
KMT2C
KMT2D
KMT2F
KMT2G
incubation time (hrs)
C
H3K27me3 KDM
KDM6B
KDM6A
H3K4me3 KDM
KDM5A
KDM5B
KDM5C
JKDM5D
D
norm
hypox
reox
0
8
24
+8
(hrs)
MCF7
MLL1/KMT2A
MLL4/KMT2B
SETD1A/KMT2F
EZH2/KMT6A
JMJD3/KDM6B
Tub
bAct
E
DMSO
UNC1999
GSK343
0 norm
24 norm
24 hypox
HIF1α
CA9
H3K27me3
Tub
H3K27me3 / Tubulin

Figure S2

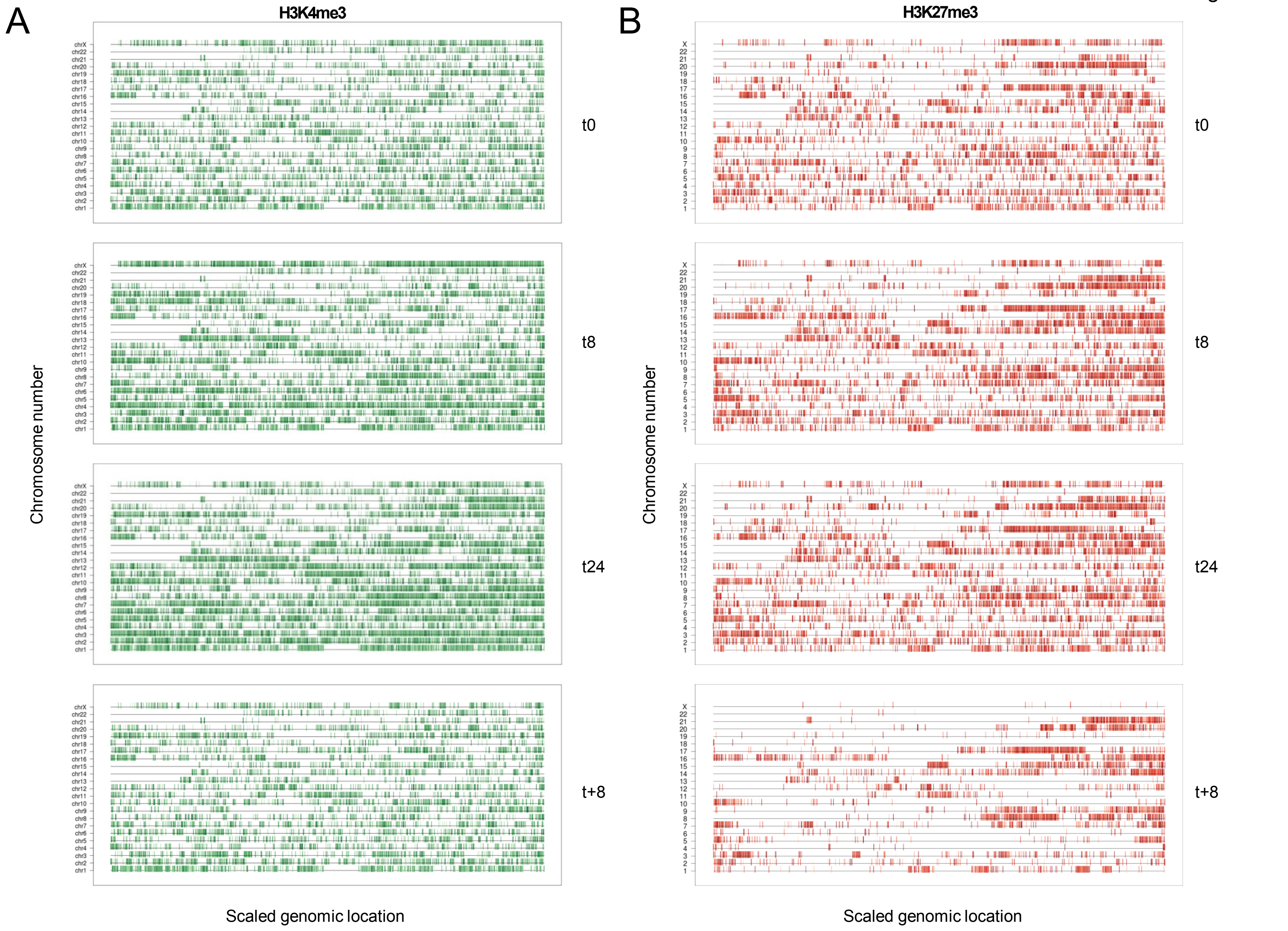
A
H3K4me3
B
H3K27me3
chrX
chr22
chr21
chr20
chr19
chr18
chr17
chr16
chr15
chr14
chr13
chr12
chr11
chr10
chr9
chr8
chr7
chr6
chr5
chr4
chr3
chr2
chr1
t0
t8
t24
t+8
Chromosome number
Scaled genomic location

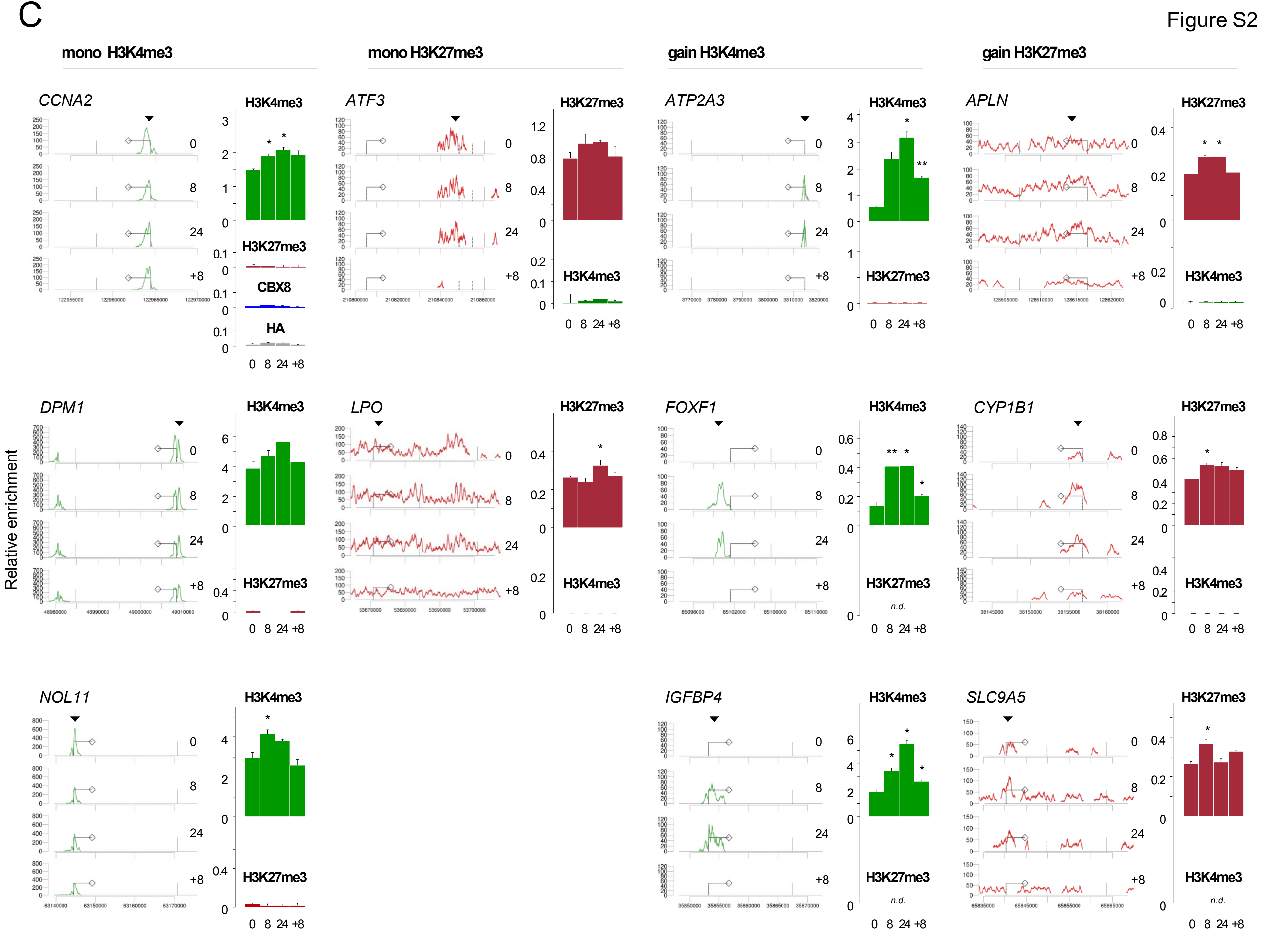
C
mono H3K4me3
mono H3K27me3
gain H3K4me3
gain H3K27me3
CCNA2
ATF3
ATP2A3
APLN
DPM1
LPO
FOXF1
CYP1B1
NOL11
IGFBP4
SLC9A5
H3K4me3
H3K27me3
CBX8
HA
Relative enrichment
0 8 24 +8
n.d.

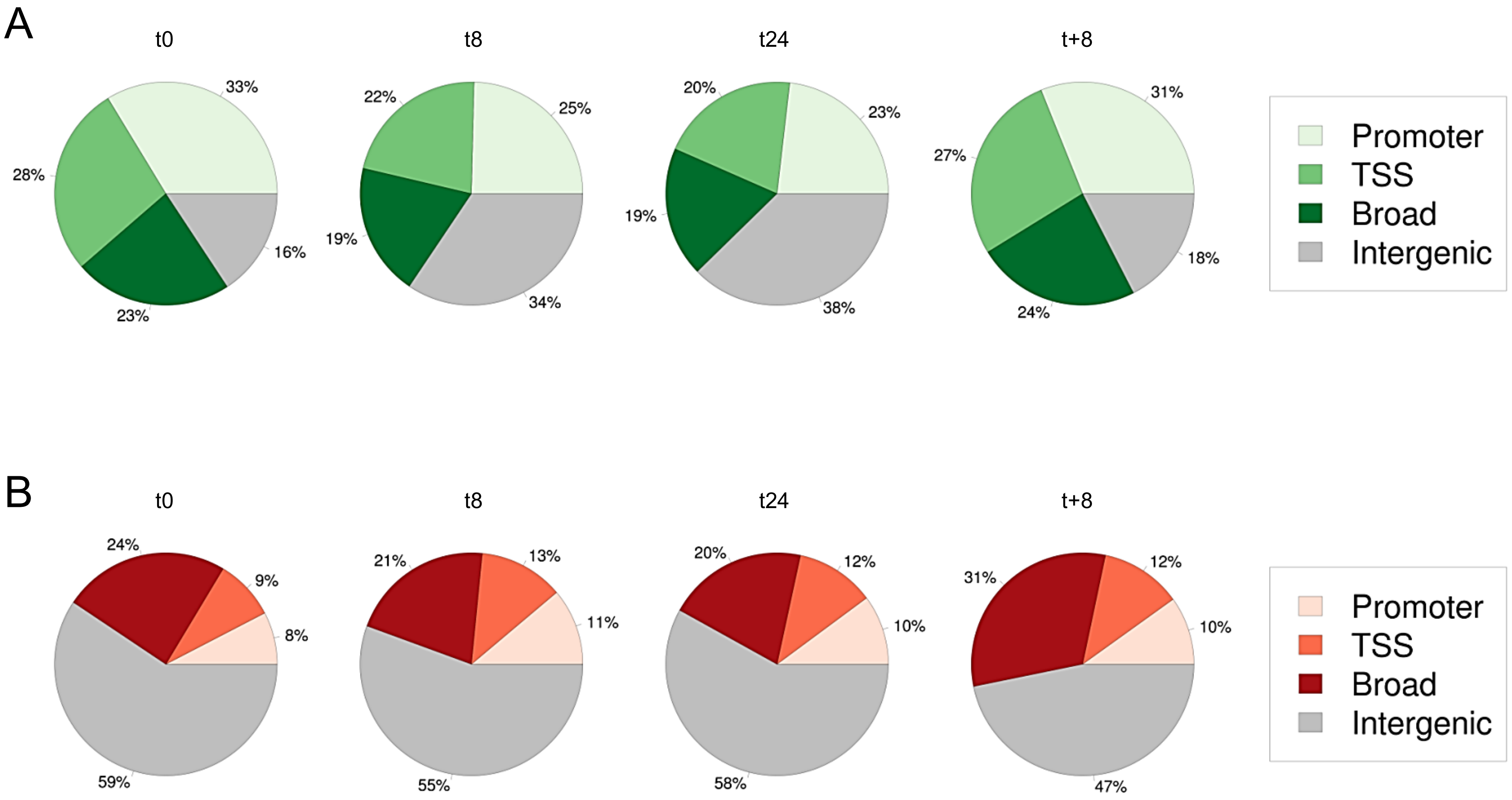
A
t0
33%
28%
16%
23%
t8
22%
25%
19%
34%
t24
20%
23%
19%
38%
t+8
31%
27%
18%
24%
Promoter
TSS
Broad
Intergenic
B
t0
24%
9%
8%
59%
t8
21%
13%
11%
55%
t24
20%
12%
10%
58%
t+8
31%
12%
10%
47%
Promoter
TSS
Broad
Intergenic

Figure S4

A

	all genes	HIF1A targets
expressed during hypoxia	11% (2544 of 22732)	30% (58 of 206)
upregulated during hypoxia	43% (325 of 751)	97% (39 of 40)

C

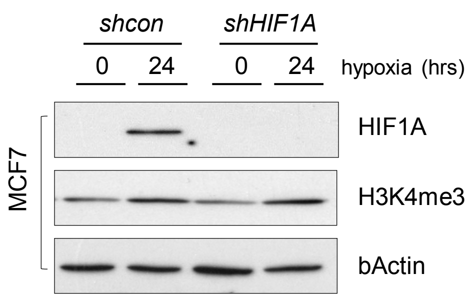

B

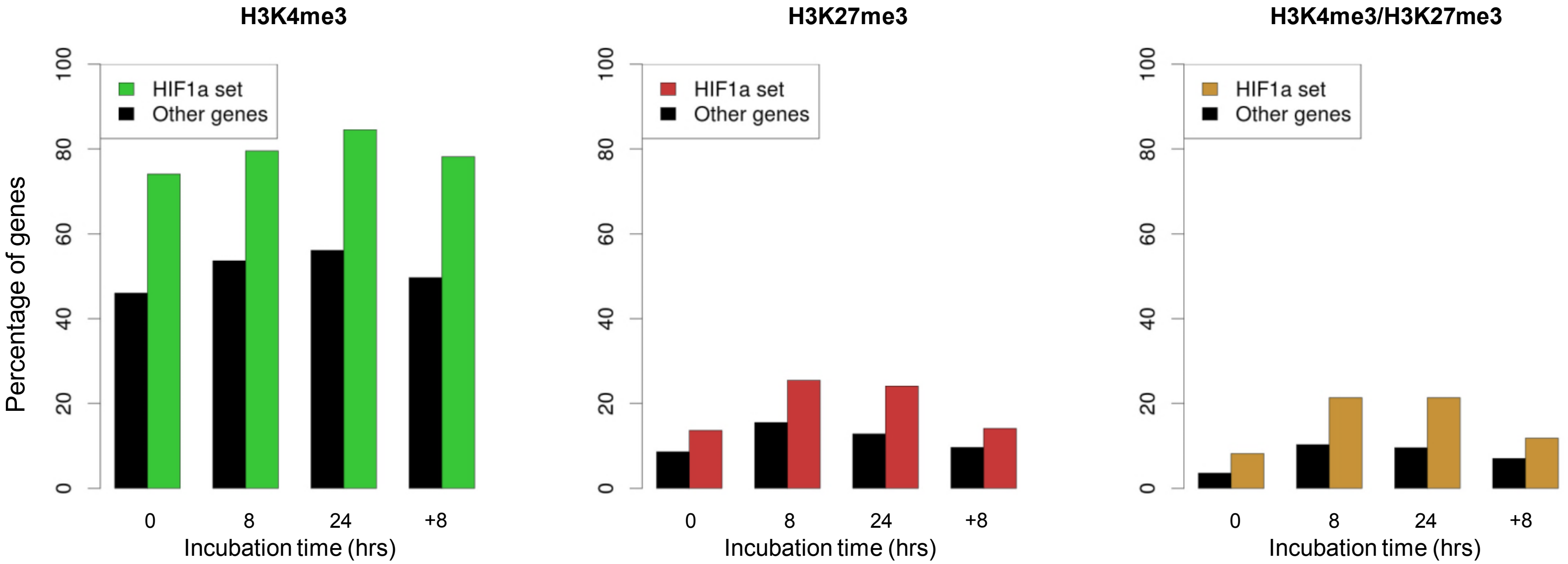

Figure S5

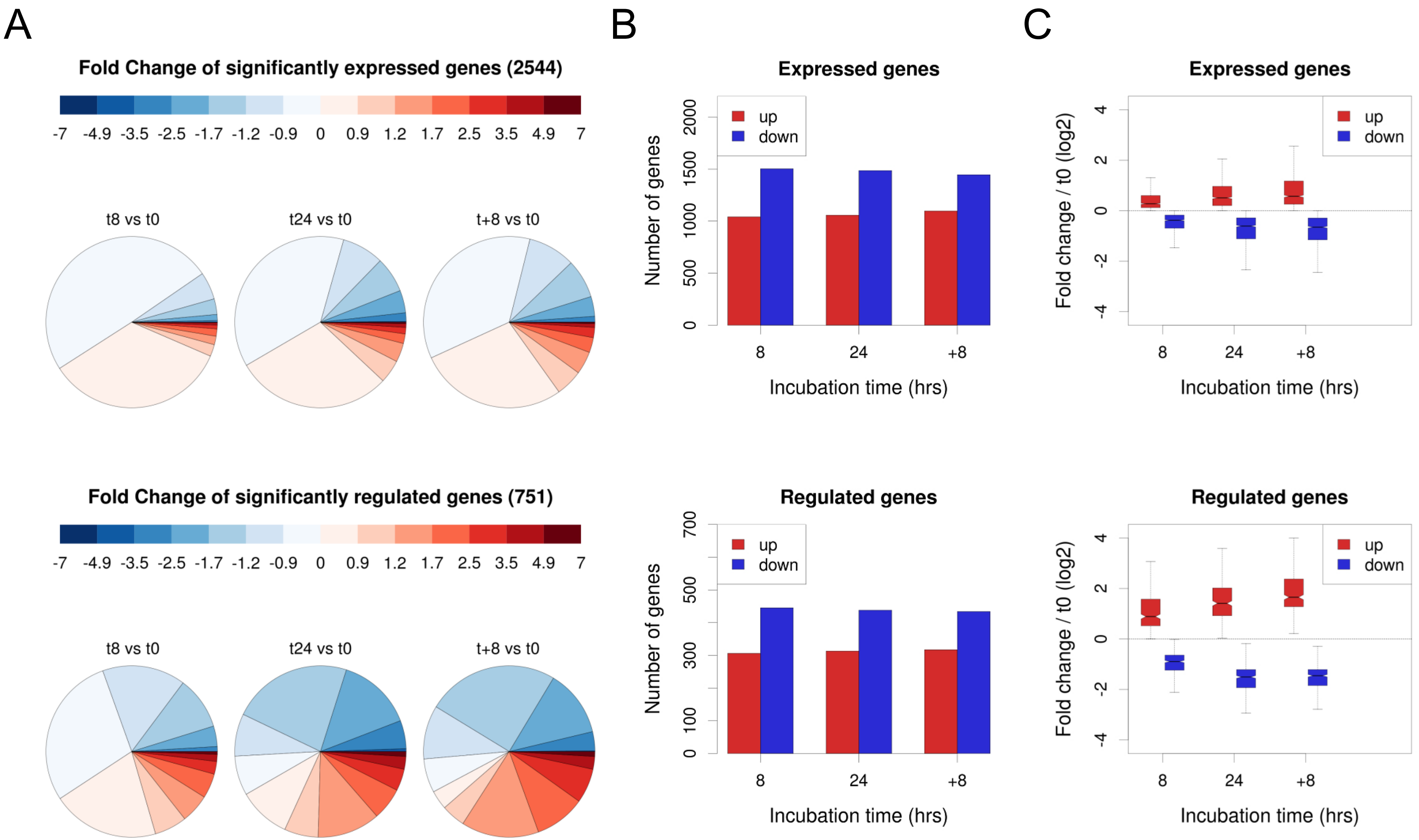
A
Fold Change of significantly expressed genes (2544)
-7 -4.9 -3.5 -2.5 -1.7 -1.2 -0.9 0 0.9 1.2 1.7 2.5 3.5 4.9 7
t8 vs t0
t24 vs t0
t+8 vs t0
Fold Change of significantly regulated genes (751)
-7 -4.9 -3.5 -2.5 -1.7 -1.2 -0.9 0 0.9 1.2 1.7 2.5 3.5 4.9 7
t8 vs t0
t24 vs t0
t+8 vs t0
B
Expressed genes
Number of genes
up
down
0 500 1000 1500 2000
8 24 +8
Incubation time (hrs)
Regulated genes
Number of genes
up
down
0 100 300 500 700
8 24 +8
Incubation time (hrs)
C
Expressed genes
Fold change / t0 (log2)
up
down
-4 -2 0 2 4
8 24 +8
Incubation time (hrs)
Regulated genes
Fold change / t0 (log2)
up
down
-4 -2 0 2 4
8 24 +8
Incubation time (hrs)

Figure S5

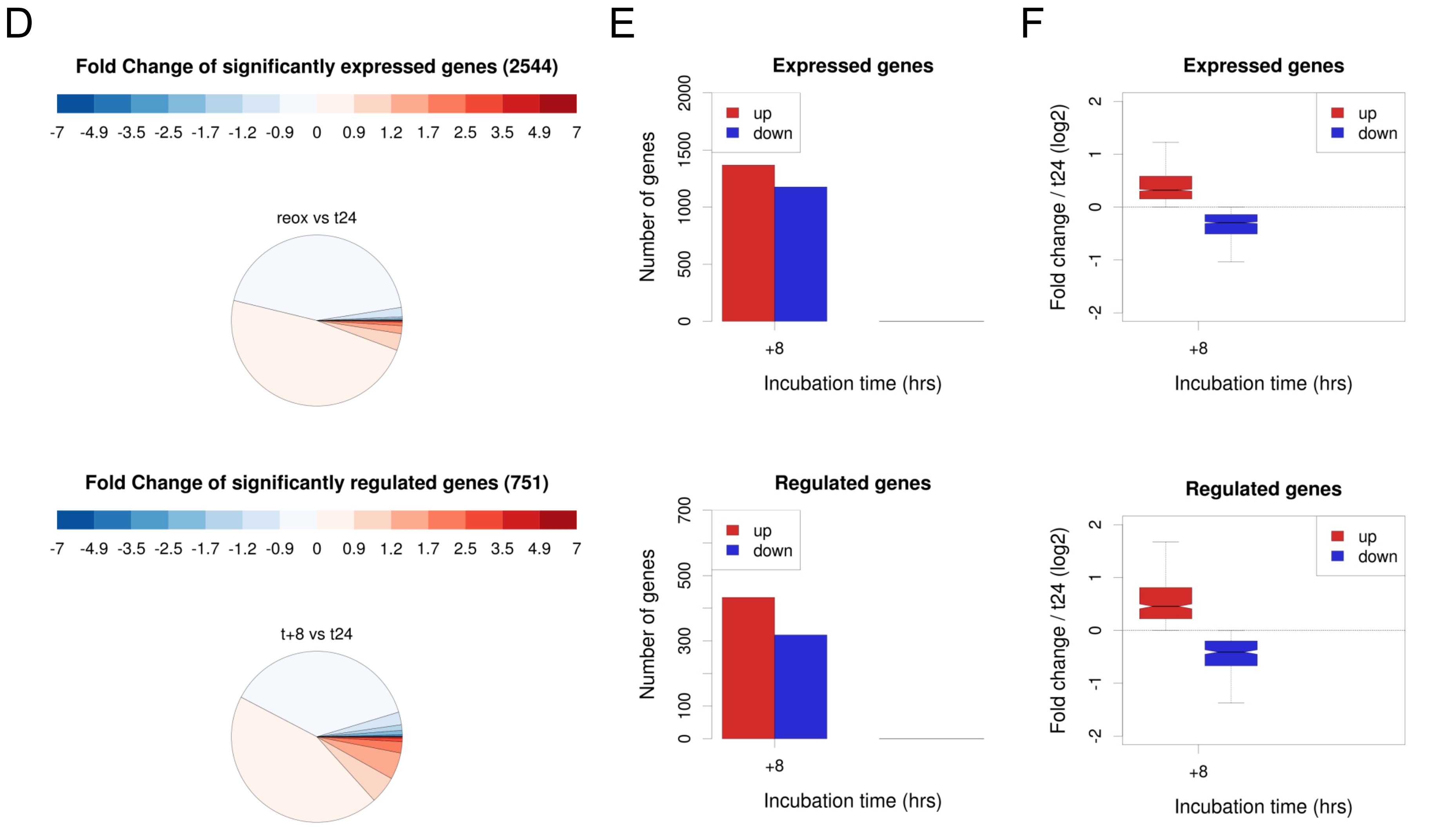
D
Fold Change of significantly expressed genes (2544)
-7 -4.9 -3.5 -2.5 -1.7 -1.2 -0.9 0 0.9 1.2 1.7 2.5 3.5 4.9 7
reox vs t24
Fold Change of significantly regulated genes (751)
-7 -4.9 -3.5 -2.5 -1.7 -1.2 -0.9 0 0.9 1.2 1.7 2.5 3.5 4.9 7
t+8 vs t24
E
Expressed genes
Number of genes
0 500 1000 1500 2000
up
down
+8
Incubation time (hrs)
Regulated genes
Number of genes
0 100 300 500 700
up
down
+8
Incubation time (hrs)
F
Expressed genes
Fold change / t24 (log2)
-2 -1 0 1 2
up
down
+8
Incubation time (hrs)
Regulated genes
Fold change / t24 (log2)
-2 -1 0 1 2
up
down
+8
Incubation time (hrs)

Figure S6

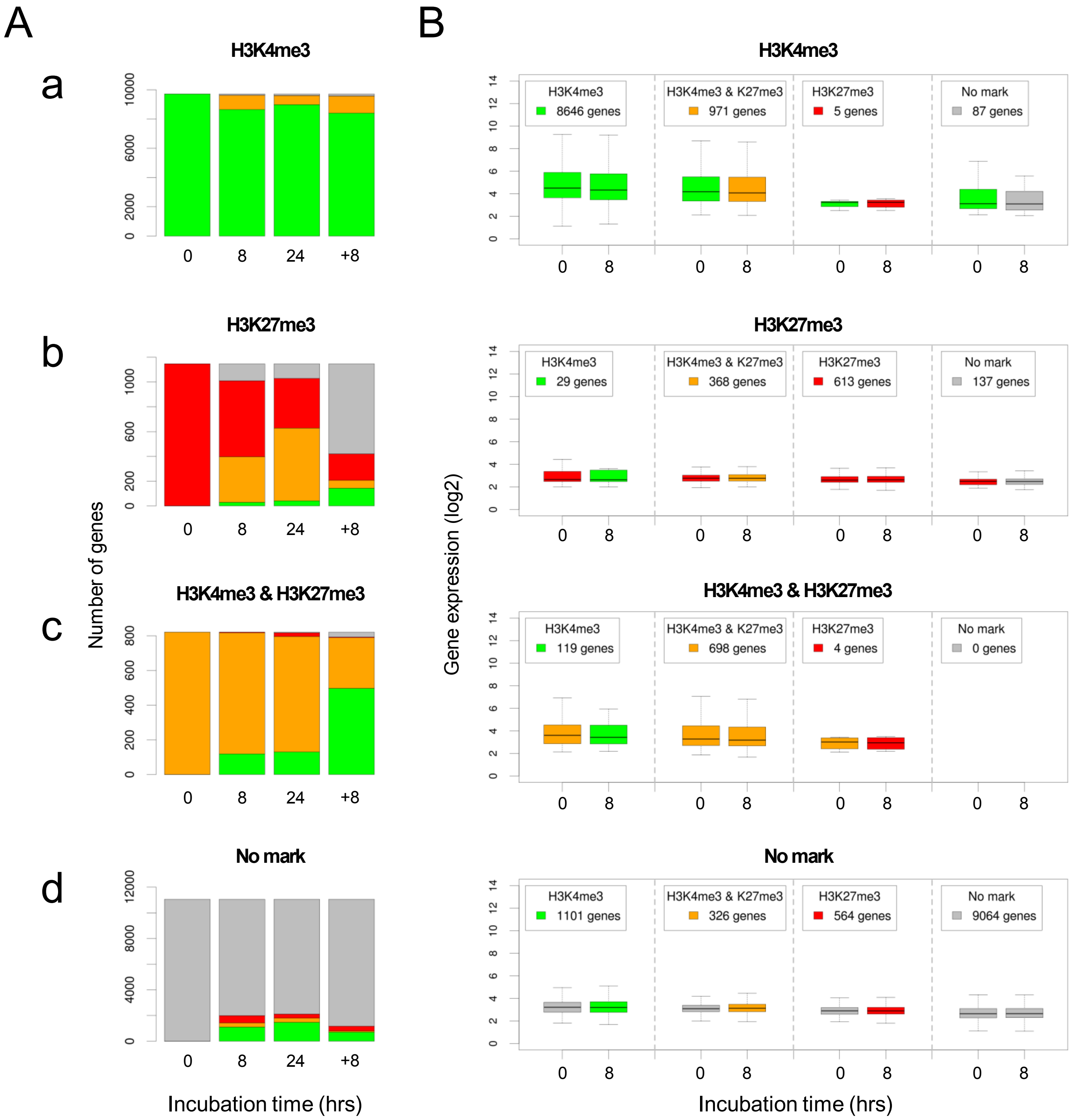

A
B
a
b
c
d
H3K4me3
H3K27me3
H3K4me3 & H3K27me3
No mark
Number of genes
Gene expression (log2)
Incubation time (hrs)
0
8
24
+8
H3K4me3
8646 genes
H3K4me3 & K27me3
971 genes
H3K27me3
5 genes
No mark
87 genes
29 genes
368 genes
613 genes
137 genes
119 genes
698 genes
4 genes
0 genes
1101 genes
326 genes
564 genes
9064 genes

Figure S6

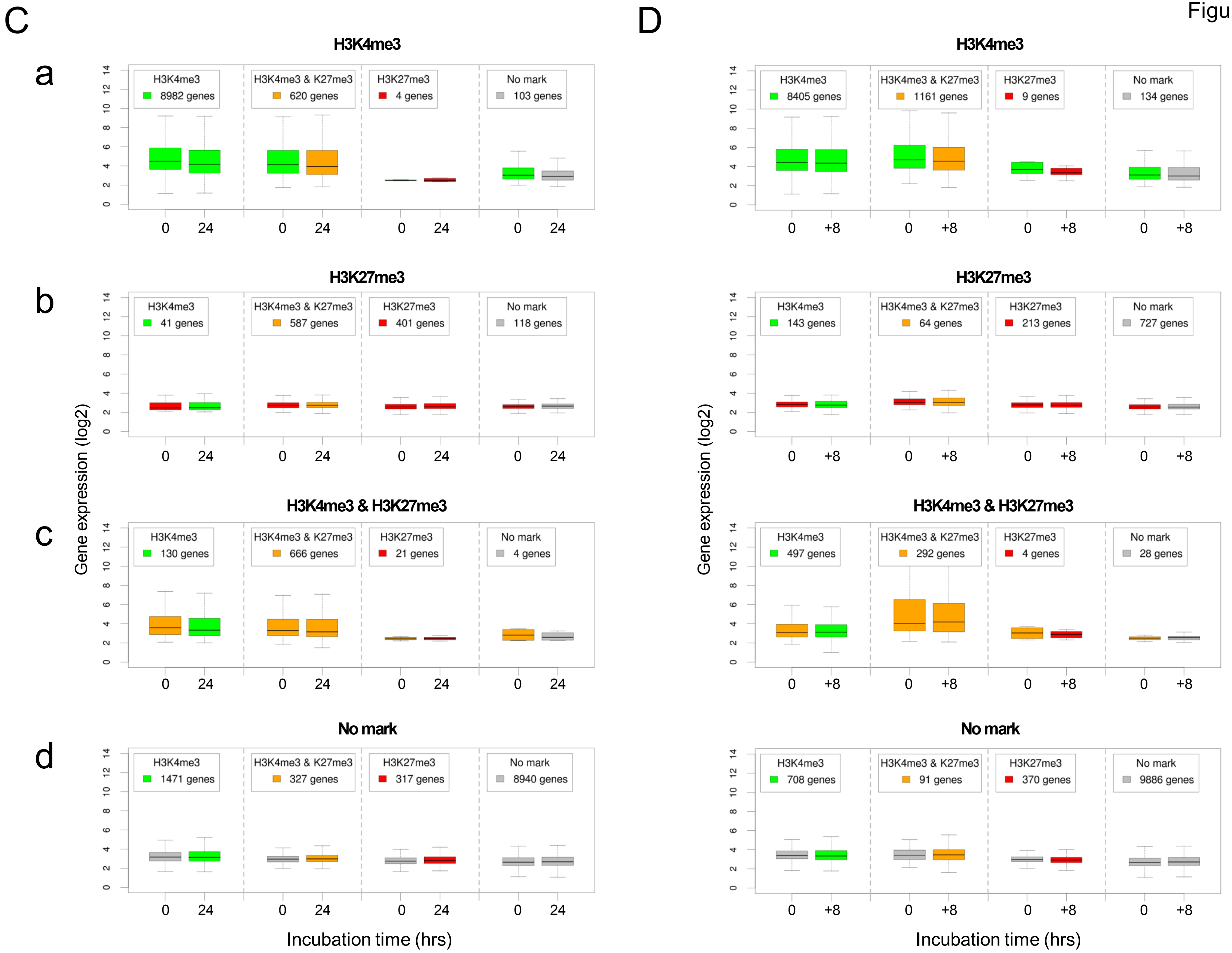
C
D
H3K4me3
H3K27me3
H3K4me3 & H3K27me3
No mark
a
b
c
d
H3K4me3
8982 genes
H3K4me3 & K27me3
620 genes
H3K27me3
4 genes
No mark
103 genes
41 genes
587 genes
401 genes
118 genes
130 genes
666 genes
21 genes
4 genes
1471 genes
327 genes
317 genes
8940 genes
8405 genes
1161 genes
9 genes
134 genes
143 genes
64 genes
213 genes
727 genes
497 genes
292 genes
4 genes
28 genes
708 genes
91 genes
370 genes
9886 genes
Gene expression (log2)
Incubation time (hrs)
0
24
+8

Figure S6

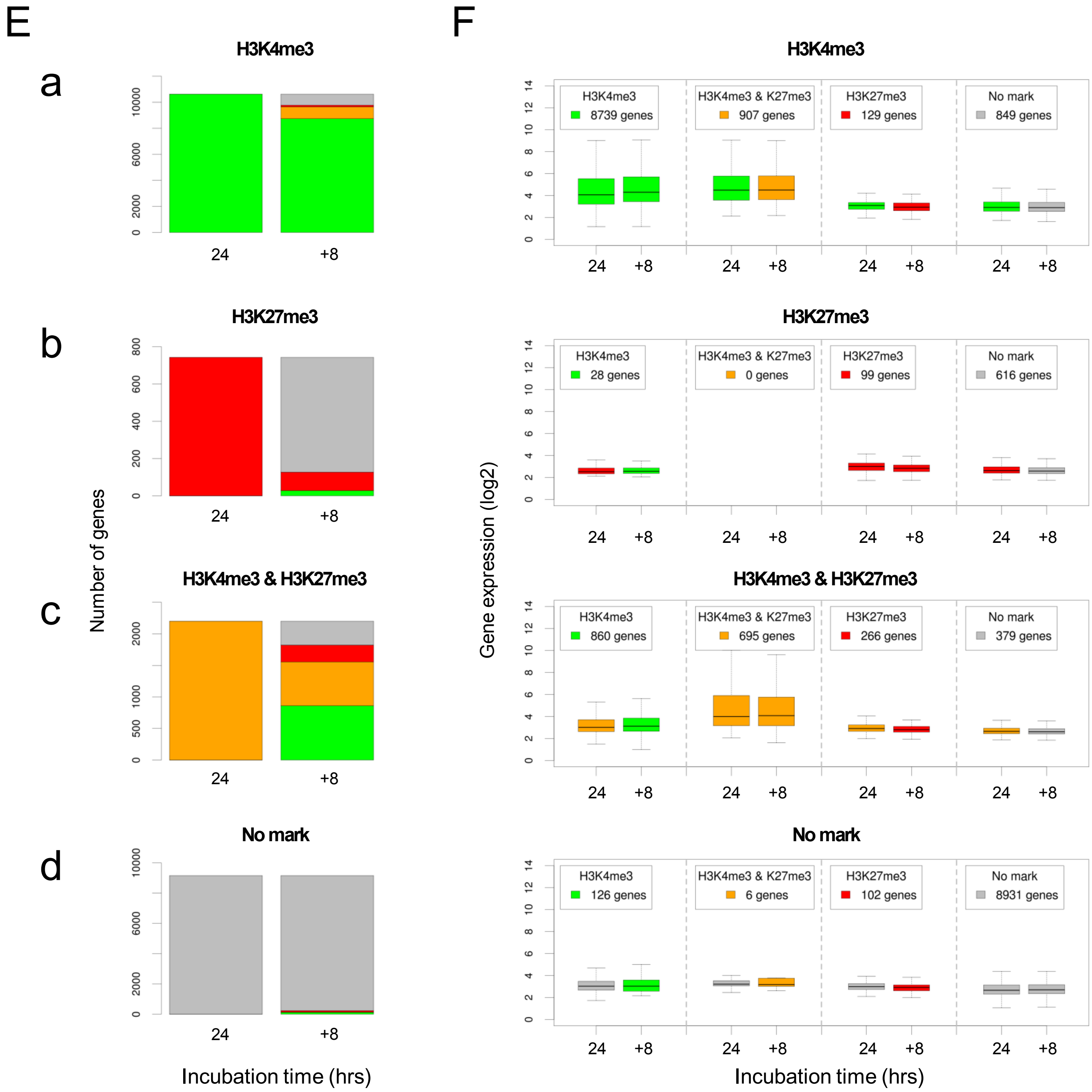
E
F
a
b
c
d
H3K4me3
H3K27me3
H3K4me3 & H3K27me3
No mark
Number of genes
Gene expression (log2)
Incubation time (hrs)
24
+8
H3K4me3
8739 genes
H3K4me3 & K27me3
907 genes
H3K27me3
129 genes
No mark
849 genes
28 genes
0 genes
99 genes
616 genes
860 genes
695 genes
266 genes
379 genes
126 genes
6 genes
102 genes
8931 genes

Figure S7

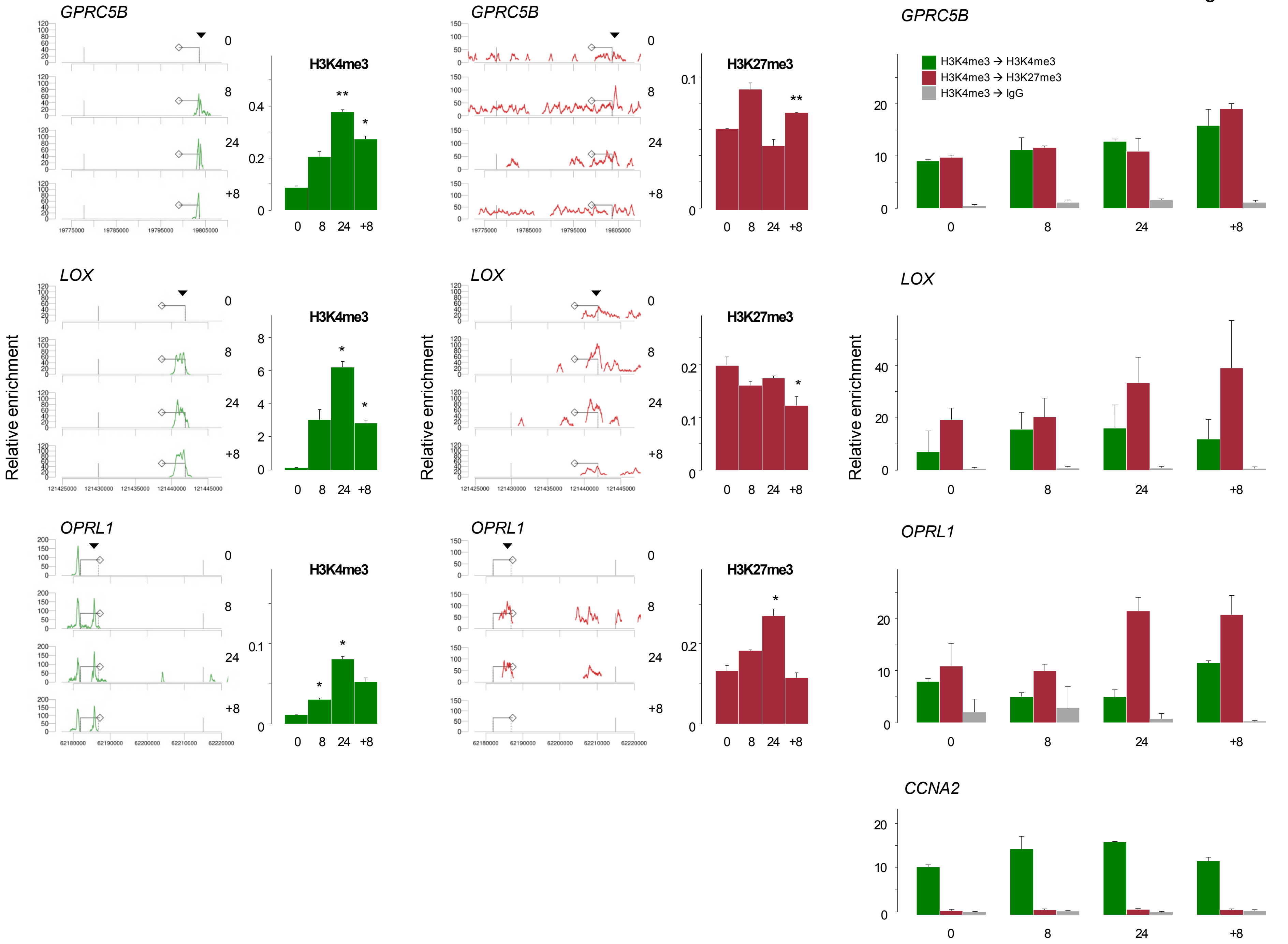

GPRC5B
H3K4me3
H3K27me3
H3K4me3 → H3K4me3
H3K4me3 → H3K27me3
H3K4me3 → IgG
LOX
OPRL1
CCNA2
Relative enrichment
0
8
24
+8

Figure S8

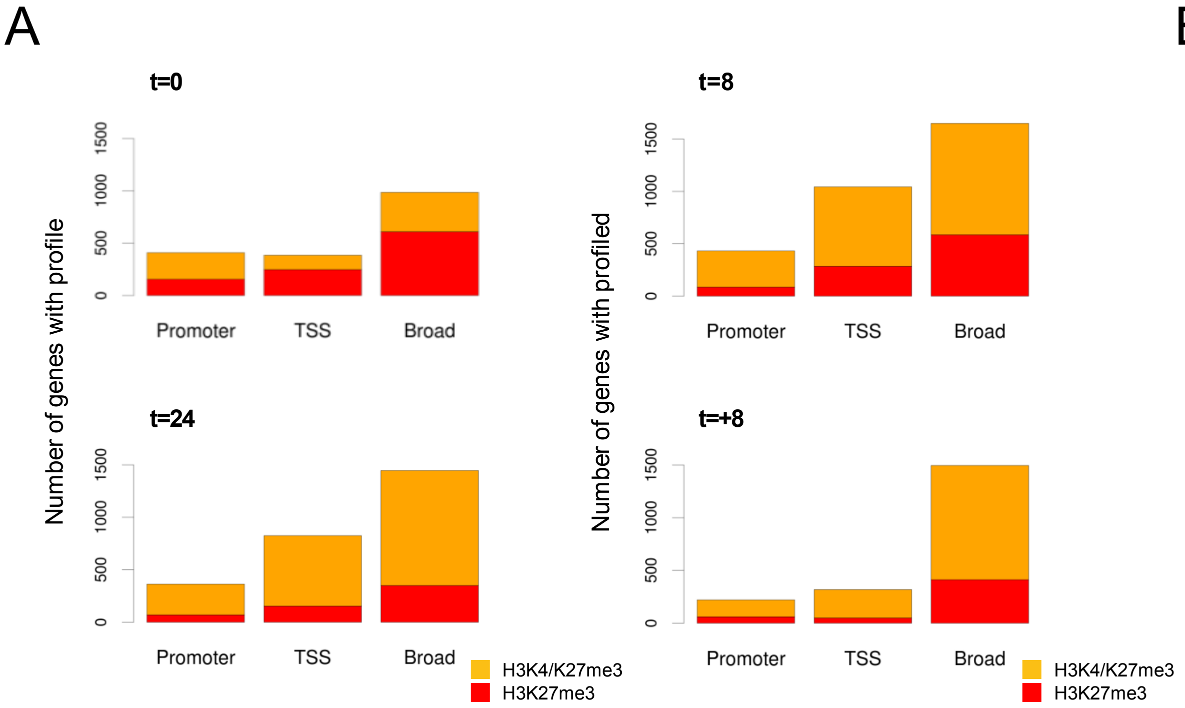
A
t=0
t=8
t=24
t=+8
Number of genes with profile
Number of genes with profiled
0
500
1000
1500
Promoter
TSS
Broad
H3K4/K27me3
H3K27me3

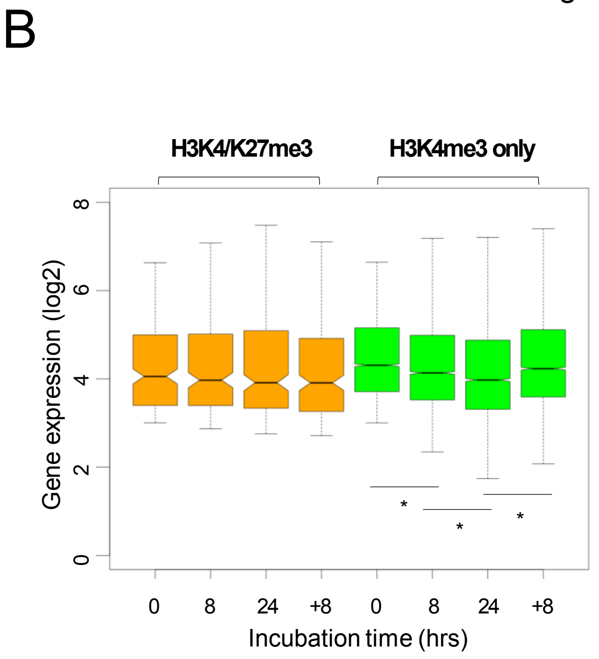
B
H3K4/K27me3
H3K4me3 only
Gene expression (log2)
0
2
4
6
8
0
8
24
+8
Incubation time (hrs)
*
