# Supplementary material for: Hypoxia increases genome-wide bivalent epigenetic marking by specific gain of H3K27me3
Source: Epigenetics Chromatin. 2016 Oct 26;9:46. doi: 10.1186/s13072-016-0086-0 (PMC5080723; doi:10.1186/s13072-016-0086-0)
Supplement: Supplementary file 2 — Additional file 2: Additional tables. [file 13072_2016_86_MOESM2_ESM.docx]

Hypoxia increases genome-wide bivalent epigenetic marking by specific gain of H3K27me3

Peggy Prickaerts *et al.*

Figure legends

**Figure S1.** Reversible, oxygen-dependent global changes of H3K4me3 and H3K27me3 levels. (*A*) Immuno-blot detection (IB) of epigenetic changes (H3K27me3 and K3K9K14ac) in MCF7 breast cancer cells and DU145 human prostate cancer cells at the indicated time points. Beta-Actin (bAct) was used as loading control. mRNA Expression profiles of (*B*) known and putative H3K4 and H3K27-methyl transferases (KMT) (*C*), known and putative H3K4 and H3K27 demethylases (KDM) and (*D*) corresponding protein expression profiles of in response to oxygen fluctuation. Alpha-Tubulin (Tub) and beta-Actin were used as loading controls. (*E*) Inhibition of EHZ2 using UCN1999 or GSK343. Left panel: representative immuno-blot detection of indicated proteins; induction of HIF1A and CA9 confirm hypoxic status. Tubulin was used as loading control. Right panel: quantification of global H3K27me3 levels. White bar: normoxic control (t=0); grey bars: normoxic references (t=24; DMSO, UCN1999, GSK343); black bars: hypoxic cells (t=24; DMSO, UCN1999, GSK343). Asterisk indicates statistical significance (*p*<0.05).

**Figure S2.** Reversible, oxygen-dependent global changes of H3K4me3 and H3K27me3 levels. Genome-wide presentation of (*A*) H3K4me3 and (*B*) H3K27me3 enrichment/peak locations at indicated experimental time points. (*C*) ChIP-PCR validation of representative, called epigenetic profiles: gene tracks (shown at left side for each locus) and ChIP-PCR bar plots (shown at right side for each locus) for the indicated loci: *CCNA2*, *DPM1*, *NOL11* for H3K4me3-only marked genes (mono H3K4me3), *ATF3*, *LPO* for H3K27me3-only marked genes (mono H3K27me3), *ATP2A3*, *FOXF1*, *IGFBP4* for genes that gained H3K4me3-marking during hypoxia (gain H3K4me3) and *APLN*, *CYP1B1*, *SLC9A5* for genes that gained H3K27me3-marking during hypoxia (gain H3K27me3). Antibodies used in ChIP: H3K4me3, H3K27me3 and HA (control; *CCNA2*); *n.d.*: ChIP not done. Diamond symbol indicates direction of transcription, black triangle indicates approximate positions of primer sets used for validation.

**Figure S3.** Genic and intergenic distribution of histone trimethylation during hypoxia. Graphical representation of counted occurrence of H3K4me3- or H3K27me3-marking at defined genomic regions: (*A*) H3K4me3-marks and (*B*) H3K27me3-marks at genic (TSS, promoter, gene body) and intergenic regions. Note: percentages represent relative distribution of peaks over the indicated genomic regions (*i.e.* no correspondence to numbers of marked genes).

**Figure S4.** Epigenetic and transcriptional regulation at HIF1a target genes. (*A*) Table shows percentage significantly expressed genes (at any time point) of all genes (total number: 22732) in MCF7 data set and of a set of 206 HIF1a target genes (([Ortiz-Barahona et al. 2010](#_ENREF_60)) and percentage of genes upregulated with a set of significantly regulated genes (absolute Log2-based FC>1.2) in MCF7 cells (total number significantly regulated genes: 751) and of the HIF1a target genes set (40) ([Ortiz-Barahona et al. 2010](#_ENREF_60)). (*B*) Bar graphs indicating percentage of HIF1a targets genes and other genes enriched for H3K4me3 (left), H3K27me3 (middle), or H3K4me3+H3K27me3 (right) under indicated experimental conditions (t=0, 8, 24 and +8 hrs). (*C*) immuno-blot analysis of global H3K4me3 levels in the presence or absence of HIF1A. Cellular HIF1A levels were reduced with a retroviral short hairpin (sh) construct. Beta Actin (bActin) was used as loading control.

**Figure S5.** Global expression changes in response to hypoxia and reoxygenation. (*A*) Pie charts indicating (upper panel) fold change (FC) of significantly expressed genes (n=2544) and (lower panel) fold change (FC) of significantly regulated genes (n=751) under indicated experimental conditions (t=8, t=24 hrs *vs* t=0). (*B*) Bar graphs indicating number of genes significantly up- (red) or down- regulated (blue) at t=8 and t=24 hrs *vs* t=0. (*C*) Box-plots showing fold changes (FC) median expression levels for all significantly expressed genes t=8 and t=24 hrs *vs* t=0. (*D*) Pie charts indicating (upper panel) fold change (FC) of significantly expressed genes (n=2544) and (lower panel) fold change (FC) of significantly regulated genes (n=751) under indicated experimental conditions t=+8 hrs *vs* t=0. (*E*) Bar graphs indicating number of genes significantly up- (red) or down- regulated (blue) under indicated experimental conditions t=+8 hrs *vs* t=0. (*F*) Box-plots showing fold changes (FC) median expression levels for all significantly expressed genes under indicated experimental conditions t=+8 hrs *vs* t=0. An expressed gene is defined as a gene with a minimum absolute expression of 100 (averaged across 3 replicates) at any experimental time point; a significantly regulated gene shows a FC of least 2 in response to its reference value at t=0.

**Figure S6.** Effect of hypoxia-induced bivalency on gene expression. (*A*) Bar graphs depicting hypoxia/reoxygenation-induced epigenetic changes (numbers of genes) at loci that were H3K4me3 only- (green), bivalently- (orange), H3K37me3 only- (red) or non-marked (grey) at t=0 (top to bottom: a, t=0; b, t=8; c, t=24; d, t=+8 hrs). (*B*) Box-plots indicating median expression values of genes (t=0 *vs* t=8 hrs hypoxia) that retained or acquired H3K4me3 only-marking (any color (t=0) to green (t=8): any color>green), that retained/acquired bivalency (any color>orange); that retained/acquired H3K27me3 only-marking (any color>red) or that retained/acquired no mark (any color>grey); panels are indicated from left to right, respectively. (*C*) Box-plots indicating median expression values of genes (t=0 *vs* t=24 hrs hypoxia) that retained of acquired indicated epigenetic marks; color coding as under (*B*). (*D*) Box-plots indicating median expression values of genes (t=0 *vs* t=+8 hrs reoxygenation) that retained of acquired indicated epigenetic marks; color coding as under (*B*). (*E*) Bar graphs depicting reoxygenation-induced epigenetic changes (numbers of genes) at loci that were H3K4me3 only- (green), bivalently- (orange), H3K37me3 only- (red) or non-marked (grey) at t=24 hrs hypoxia (top to bottom). (*F*) Box-plots indicating median expression values of genes (t=24 hrs hypoxia t=0 *vs* t=+8 hrs reoxygenation) that retained or acquired indicated epigenetic marks; color coding as under (*B*).

**Figure S7.** Hypoxia increases genic bivalent marking. Bioinformatic calling of enriched regions (gene tracks shown at left side for each locus) was validated by ChIP-PCR (bar plots; shown at right side for each locus) for a number of representative epigenetic profiles: *GPRC5B*, *LOX and OPRL1.* Antibodies used in ChIP: H3K4me3 (green), H3K27me3 (red). Diamond symbol indicates direction of transcription, black triangle indicates approximate position of primer set used for validation. Bar diagrams at far-right show outcome of re-ChIP experiments: Antibody combinations used in ChIP/re-ChIP: H3K4me3/H3K4me3 (green), H3K4me3/H3K27me3 (red) and H3K4me3/IgG control (grey). The single-H3K4me3-marked *CCNA2* locus (bottom panel) was used as a methodological control.

**Figure S8.** Hypoxia increases genic bivalent marking. (*A*) Bar graphs depicting H3K27me3 only-marked and bivalently–marked gene numbers at the indicated time points, differentiated by subgenic location of H3K27me3-marking (*promoter-*, *TSS-*, *broad-*profile marking). (*B*) Box-plots indicating median expression values of a select set (Log2-based expression value 3.68-6.64 at t=0) of genes that had acquired bivalent marking during hypoxia and sustained this epigenetic status upon reoxygenation; color coding: orange: H3K4me3 & H3K27me3-marked genes; green H3K4me3 only-marked genes. Asterisks (*) indicates statistical significant differences at p<0.05.

Table legends

**Table S1.** GO-analysis of genes and processes induced and down-regulated by hypoxia (t=24 hrs).

**Table S2.** GO-analysis of genes and processes induced and reduced by reoxygenation (t=+8 hrs).

**Table S3.** GO-analysis of genes and processes associated with bivalent markers at t=8, t=24 hours hypoxia; reference lists: t=0 and processes associated with known bivalent markers in embryonal stem cells ([Bernstein et al. 2006](#_ENREF_14)).

**Table S4.** Primers used for ChIP and re-CHIP analysis.

**Table S5.** Primers used for quantitative RT-PCR analysis.

Table S1

Table S2


Table S3

Table S4

**Gene Direction ChIP primer sequence (5’→3’)**

*CCNA2 (cyclin-A2)* Forward TGACGTCATTCAAGGCGACAG

Reverse GCTCAGTTTCCTTTGGTTTACCC

*ATF3 (cyclic AMP-dependent transcription factor ATF3)* Forward TGTTTTTTCTTTTGCGTTTGGC

Reverse TCGTGGCAACCAAATCTAAACAG

*ATP2A3 (sarcoplasmic/endoplasmic reticulum calcium ATPase 3)* Forward GCTCGAATTCTGCGAAGTGAC

Reverse GCTTATTCAGAGGCTTTCGGG

*APLN (apelin)* Forward AGGGCAACGAATTTATCCAAA

Reverse CGTGCTTTAATTCCCCCGT

*DPM1 (dolichol-phosphate mannosyltransferase1)* Forward GCAGATCCTGACCGCCTA

Reverse TACTTGGAAGCGAGGCTGA

*LPO (lactorperoxidase)* Forward GCTGTCAGGTCTTCATCTCATTTC

Reverse TCGGATCTGCTGCAGATGG

*IGFBP4 (insulin-like growth factor-binding protein 4)* Forward CTCTTCCTTGAACGAGTCTCCC

Reverse AGCACCAGTTCCTGGAGCTC

*SLC9A5 (sodium–hydrogen exchanger 5)* Forward GGCTTCTTGCACGGTGCT

Reverse AGCCCGGATTTGTGGAATC

*NOL11 (nucleolar protein 1)* Forward GTGGAGCAGAGCGACAAAAC

Reverse CCTGCCGCTGTCTGTCACTA

*CYP1B1 (cytochrome P450 1B1)* Forward GACTGAGTGCCGTTGGGTG

Reverse CGTCTTCGGCCATTTCTCC

*FOXF1 (forkhead box protein F1)* Forward GTGCACACACGCATGGGT

Reverse CCCGGTAAATTACGCAAACA

*KLK11 (kallikrein-11)* Forward ATGGAGCTGAAGTTTGTGCTGA

Reverse AATCCATCTCTTCCCCTGTTCC

**Gene Direction Re-ChIP primer sequence (5’→3’)**

*LOX (lysyl oxidase)* Forward CGCTGAGGCTGGTACTGTGA

Reverse AGAACAACGGGCAGGTGTTC

*OPRL1 (opioid related nociceptin receptor 1)* Forward GCCCCTCCTTAGCTCCTG

Reverse AGGGGGTGTAGGGACCAA

*GPRC5B (G protein-coupled receptor class C group 5 member B)* Forward TCATAAGCAGTAACTTCCCCGAA

Reverse CTGCGTCCTGGACCTGGT

Table S5

**Gene Category Direction Primer sequence (5’→3’)**

*ADCY5 (adenylate cyclase 5)* gain H3K27me3 Forward TCTGAGCACGTCCGCAAGT

Reverse CAAATCGGTCGTCTACCTGCTT

*AKAP1 (A-kinase anchoring protein 1)* gain H3K27me3 Forward CGTGGACTACGGCGGATATAA

Reverse AAACGGCAGGGTGACAAAGT

*ARRB1 (arrestin beta 1)* gain H3K27me3 Forward CGACGTGGCCGTGGAA

Reverse TTGGTATCTACTGGCGTCTCGTT

*ASB8 (ankyrin repeat and SOCS box containing 8)* mono H3K4me3 Forward TGTCTGGGAAAATCAAAGAGAGTTAC

Reverse CTCTTACGCATCCCCAGAACA

*FAM217B (family with sequence similarity 217 member B)* gain H3K27me3 Forward AGCAGCTCTAAGGTGGAAACCA

Reverse GTGGCATGACCACAGCTCTCT

*LRRC28 (leucine rich repeat containing 28)* mono H3K4me3 Forward TCCCTTGAGAGAGACGCCAAT

Reverse GAGCAGCAGTAAGCCACAAAACT

*PPAT (phosphoribosyl pyrophosphate amidotransferase)* mono H3K4me3 Forward TGCTCTTGCTTACGCAGGAA

Reverse TGTTTGGCTGAATGAAGGTTCTC

*RPL31P17 (ribosomal protein L31 pseudogene 17)* mono H3K4me3 Forward GACACCAGGCTCAACAAAGCT

Reverse GCCGCACACGGATTCG

*ACTB (actin beta)* reference Forward CCTGGCACCCAGCACAAT

Reverse GCCGATCCACACGGAGTACT

*PPIA (cyclophilin A)* reference Forward TTCCTGCTTTCACAGAATTATTCC

Reverse GCCACCAGTGCCATTATGG
